# Bumblebees land rapidly by intermittently accelerating and decelerating toward the surface during visually guided landings

**DOI:** 10.1016/j.isci.2022.104265

**Published:** 2022-04-16

**Authors:** Pulkit Goyal, Johan L. van Leeuwen, Florian T. Muijres

**Affiliations:** 1 Experimental Zoology Group, Wageningen University and Research, P.O. Box 338, 6700 AH, Wageningen, the Netherlands

**Keywords:** biological sciences, zoology, ethology, sensory neuroscience

## Abstract

Many flying animals parse visual information to control their landing, whereby they can decelerate smoothly by flying at a constant radial optic expansion rate. Here, we studied how bumblebees (*Bombus terrestris*) use optic expansion information to control their landing, by analyzing 10,005 landing maneuvers on vertical platforms with various optic information, and at three dim light conditions. We showed that bumblebees both decelerate and accelerate during these landings. Bumblebees decelerate by flying at a constant optic expansion rate, but they mostly accelerate toward the surface each time they switched to a new, often higher, optic expansion rate set-point. These transient acceleration phases allow bumblebees to increase their approach speed, and thereby land rapidly and robustly, even in dim twilight conditions. This helps explain why bumblebees are such robust foragers in challenging environmental conditions. The here-proposed sensorimotor landing control system can serve as bio-inspiration for landing control in unmanned aerial vehicles.

## Highlights

• Bumblebees land by intermittently decelerating and accelerating toward a surface
• Acceleration and deceleration phases result from a single visual-motor controller
• The accelerations toward the surface allow bees to maximize their landing speed
• Bumblebees adjust their sensorimotor control response to fly slower in dim light

## Graphical abstract

**Figure:**
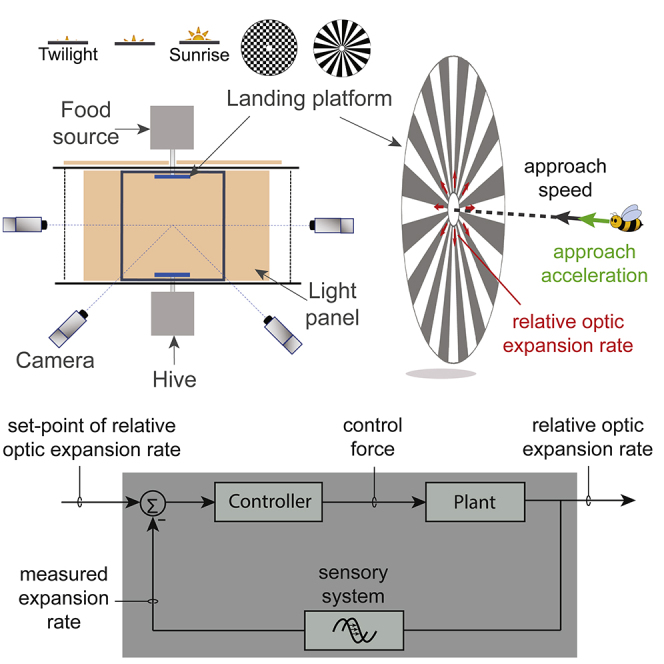

## Introduction

Bumblebees rely on the landing phase of their flight to visit flowers and gather nectar and pollen, and thus the ability to land rapidly and precisely is essential for their survival and reproduction. Foraging bumblebees can visit flowers at very high frequencies, with up to 1000 flower visits in a single hour (Heinrich, 1979). For each visit, they accurately control their flight speed so that it reduces to near zero close to the landing surface (Chang et al., 2016; Reber et al., 2016a). Such accurate control ensures soft touchdown and thus reduces the risk of damage that can be caused by high-impact collisions (Foster and Cartar, 2011; Mountcastle and Combes, 2014; Rajabi et al., 2020).

Similar to many other flying animals (Lee et al., 1991, 1993; Srinivasan et al., 2000; Van Breugel and Dickinson, 2012; Baird et al., 2013; Liu et al., 2019; Tichit et al., 2020b), bumblebees use visual information to accurately control their flight speed during landing (Chang et al., 2016; Goyal et al., 2021a). As they advance toward a surface for landing, their motion relative to the landing surface generates expanding optical flow in which various features in the visual field appear to move radially outward from the point that is being approached (Gibson, 1955; Edwards and Ibbotson, 2007). Bumblebees may use these optical expansion information along with the retinal size of an object (Wagner, 1982) or angular position of features in the visual field (Baird et al., 2013) to measure the relative rate of expansion *r* or its inverse, the optical variable τ (τ = 1/*r*, referred to as parameter ''time-to-contact'' in literature) (Lee, 1976; Gibson, 1986). Note that insects also use optic flow cues to exhibit behaviors other than landing such as controlling both their height and flight-speed in open spaces, increasing their distance from the oncoming obstacle or observing centering behavior while flying in the narrow corridors. For reviews, see Srinivasan (2011a, 2011b), Serres and Ruffier (2017) and Baird et al. (2020).

**Figure 1:**
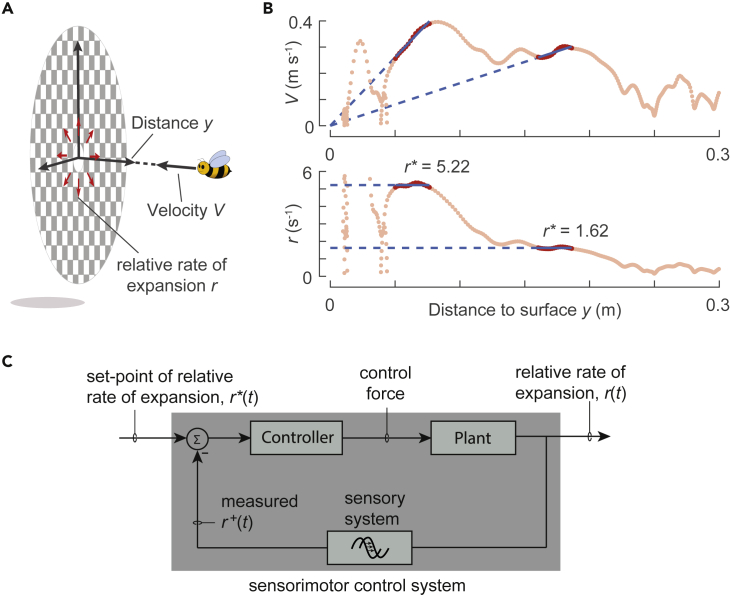
**The landing strategy of bumblebees and hypothesized sensorimotor control system that bumblebees may use during landing** (A) A schematic of a bumblebee approaching a landing surface. Due to its motion perpendicular to the landing surface, the bumblebee flying at an approach velocity *V* and at a distance *y* experiences a relative optical rate of expansion *r* = *V*/*y*. (B) The variation of approach velocity *V* and relative rate of expansion *r* with perpendicular distance from the platform *y* for a typical landing maneuver of a bumblebee. The red sections show the periods when the landing bumblebee keeps the relative rate of expansion approximately constant. The constant *r* values are referred to as set-points of the relative rate of expansion *r*∗ and are indicated by the dashed blue lines (as slope and ordinate values in the *V*-*y* and *r*-*y* graphs, respectively). The black arrow indicates the data reading direction as a bumblebee approaches the landing platform. (C) The closed-loop sensorimotor control system that we hypothesize bumblebees use during landing. As a bumblebee approaches a landing surface, the relative rate of expansion measured by the sensory system is compared with the desired set-point to generate an error-input for the controller; the controller would then convert this input into changes in the body and wing beat kinematics to modulate aerodynamic forces (control forces) that act on the animal (represented as plant). This way, the expansion rate *r* converges toward the preferred set-point value *r*^∗^. Note that the “plant” subsystem represents the dynamics of motion of the animal and the computation of relative rate of expansion as its output. This feedback control loop is similar to the forward flight speed controller in *Drosophila*, based on front-to-back optic flow (Fry et al., 2009; Rohrseitz and Fry, 2011; Medici and Fry, 2012).

The relative rate of expansion *r* or the optical variable τ are both measures of the remaining time before collision (time-to-contact) without requiring any explicit measurement of the relative distance of the animal from the object or the speed of the animal (Lee, 1976; Gibson, 1986). From a pure viewpoint of the Gibson’s framework, these perceptual variables can be measured directly by an animal’s visual system (including human's) to estimate the time-to-contact (Gibson, 1986; Serres and Ruffier, 2017; Dauxère et al., 2021). The relative rate of expansion *r* or the optical variable τ encodes the ratio of approach velocity *V* and distance to the landing surface *y* as *r* = *V*/*y* or τ = *y*/*V* (Figure 1A).

In our previous study on the landing dynamics of bumblebees (*Bombus terrestris*), we investigated how bumblebees use this relative rate of expansion to decrease their approach velocity as they reach closer to the landing surface (Goyal et al., 2021a). This showed that bumblebees tend to decelerate in multiple bouts during their landing approach. During each bout, a bumblebee flies by keeping the relative rate of expansion approximately constant; this constant is referred to as a set-point of relative rate of expansion. From one bout to the next, bumblebees tend to increase this set-point (Figure 1B).

Also other studies have considered the landing strategies of honeybees (Srinivasan et al., 2000; Baird et al., 2013), bumblebees (*Bombus impatiens*) (Chang et al., 2016), fruit flies (Van Breugel and Dickinson, 2012; Baird et al., 2013), pigeons (Lee et al., 1993), hummingbirds (Lee et al., 1991), and mallard ducks (Whitehead, 2020). These studies describe the general landing dynamics of these animals, and some of them propose the underlying basic sensorimotor control strategies. But none of these strategies can explain how bumblebees execute their modular landing strategy. Here, we aim to unravel the high-level sensorimotor control system used by bumblebees during their modular landing approach.

To study the sensorimotor control of flying animals, principles from control theory can be used. With this theory, the complex physiological mechanisms of the sensorimotor control pathways that animals employ to exhibit a wide range of behaviors are often abstracted using a system level approach. For reviews on this approach, see Taylor et al. (2008), Cowan et al. (2014); Roth et al. (2014) and Dickinson and Muijres (2016); for individual examples, see Dickson et al. (2008), Fry et al. (2009), Rohrseitz and Fry (2011), Lehmann et al. (2012), Medici and Fry (2012), Fuller et al. (2014), Sun (2014), Stöckl et al. (2017), and Wang et al. (2017). In this context, a set-point-reaching behavior in animals is analogous to the negative feedback loops commonly applied in control engineering (Figure 1C; Ogata, 2010). Here, the measured output of a system is compared with its input, the preferred value (or a set-point), to generate an error-input for the controller. Based on this input, the controller generates an appropriate motor command so that the output of the system converges to the desired set-point.

For example, to fly at a particular forward flight speed, fruit flies (*Drosophila melanogaster*) use the front-to-back translatory optic flow (Fry et al., 2009; Rohrseitz and Fry, 2011; Medici and Fry, 2012). They compare the translatory optic flow measured by the visual sensors with its desired set-point and generate an error-input for the controller. The controller in turn converts this input into a change in wingbeat and body kinematics of the animal. These changes, in turn, produce forces that act on the animal (in control terminology “plant”) so that the translatory optic flow it is experiencing during its flight converges to its desired set-point.

**Figure 2:**
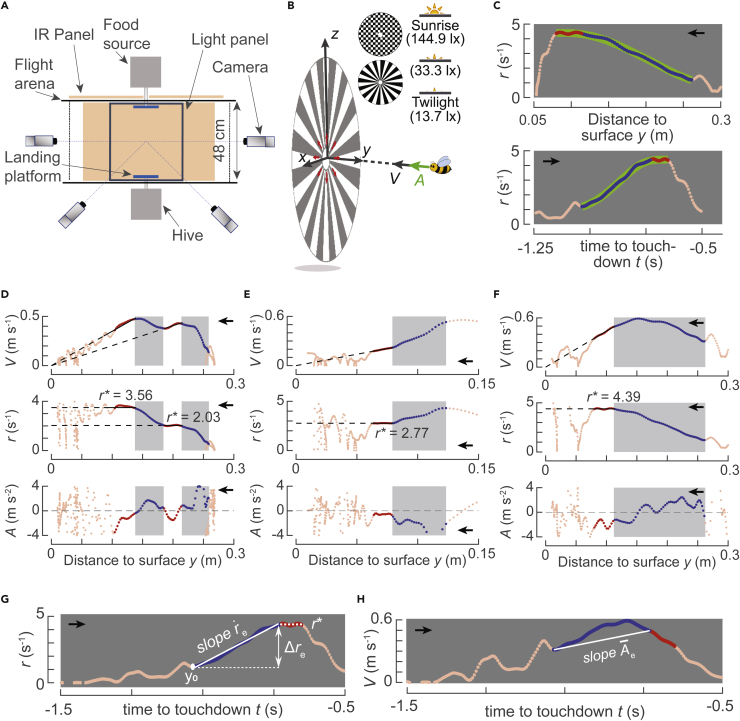
**Experimental setup and conditions, the flight kinematics of typical landing maneuvers of foraging bumblebees, and definitions of the parameters used to analyze the landing dynamics** (A) Top view of the experimental setup consisting of two vertical landing discs connected to a hive and food-source, a four-camera videography system for tracking bumblebees in real-time, and a LED light panel to vary light intensity (Goyal et al., 2021a). (B) Each landing is described in a Cartesian coordinate system with its origin at the center of the landing disc, *z* axis vertically up, and *y* axis normal to the disc and pointing into the flight arena. Four parameters describe the landing kinematics: approach distance *y*, approach velocity *V* = −*v*, approach acceleration *A* = −*a*_*y*_, and the relative rate of optical expansion *r* = *V*/*y* (red arrows). The different visual patterns on the landing disc and tested light intensities are also shown. (C) The variation of *r* with approach distance *y* and time *t* for a typical bumblebee landing maneuver. The landing maneuver includes a constant-*r* segment (red) and an entry segment directly preceding the constant-*r* segment (blue). For the entry segment and constant-*r* segment combined, we determined the low-pass filtered variation of *r*(*t*) as *r*_f_(*t*) (light green). The temporal dynamics of *r*_f_(*t*) is modeled using the closed-loop sensorimotor control system depicted in Figure 1C. (D–F) The variation of the parameter set (*V*, *A*, *r*) with *y* for landings initiated from free-flight (D, E) and take-off (F) in the presence of a spoke landing pattern and sunrise light condition. During the entry segments (blue data, highlighted in gray), bumblebees accelerate (D), decelerate (E), or do both (F) to reach their set-point of relative rate of expansion (*r*^∗^). (G and H) The entry segments are characterized using five parameters: the derivative of relative rate of expansion referred to as expansion acceleration ($\dot{r_{\text{e}}}$), the relative rate of expansion set-point (*r*^∗^), the required step change to reach the set-point (Δ*r*_e_), the initial distance from the landing platform (*y*_0_), and the average acceleration during the entry segment (${\bar{A}}_{\text{e}}$). (C–H) The black arrows indicate the direction in which abscissa data varies as a bumblebee approaches the landing disc. See also Figures S2, S9, and S10.

Here, we apply a control theoretic approach to investigate the sensorimotor control system of landing bumblebees and show how they use it to advance toward the landing surface. For this purpose, we use a database of 10,005 maneuvers of foraging bumblebees landing on a vertical platform. These maneuvers correspond to bumblebees landing on two platforms—one connected to the hive and the other to a food source. During these maneuvers, bumblebees landed directly after a take-off and from a free-flight condition (Figures 2A and 2B) (Goyal et al., 2021a). These two landing types resemble landings of bumblebees in nature that occur within short distances (e.g., within a flower patch) and long distances (e.g., between flower patches and the hive), respectively. In these landing maneuvers, the different set-points of relative rate of expansion that bumblebees flew at were identified using an algorithm described in Goyal et al. (2021a).

We hypothesize that bumblebees exhibit a set-point reaching behavior during landing, i.e. their sensorimotor control mechanism during landing uses the relative rate of expansion as a controlled variable; bumblebees use their visual system to obtain this relative rate of expansion as a sensory measurement and produce accelerations using their flight motor system to converge toward the set-point of the relative rate of expansion. To provide evidence in the support of this hypothesis, we study the overall closed-loop response of bumblebees as they transitioned from one constant set-point to a higher one. Specifically, we analyze the track segments before they reached the set-points, hereafter referred to as *entry* segments, during which bumblebees accelerate (Figure 2D), decelerate (Figure 2E), or both (Figure 2F) to reach the set-point. Using a system identification approach from control theory, we show that the observed time course of relative rate of expansion in the track segments leading up to the set-points is the transient response of the sensorimotor control system that is aiming to reach the desired set-points. This suggests that landing bumblebees use a sensorimotor control loop that is based on the relative rate of expansion *r*.

To also understand how bumblebees use the transient response of this *r*-based control loop to advance toward the landing platform, we first characterize their transient response as a motion at a constant expansion-acceleration ($\dot{r}$). The expansion-acceleration is the time-derivative of relative rate of expansion and defines how fast the animal increases or decreases its relative rate of expansion. Using this expansion-acceleration, we then identify how bumblebees regulate their transient response. This shows that the regulation of the transient response helps bumblebees to intermittently accelerate toward the surface during their landing approach. These surprising acceleration phases allow bumblebees to land more rapidly than if they would perform nonaccelerated landings.

We also test how environmental conditions affect the transient response of the sensorimotor control system of bumblebees by analyzing their landing maneuvers from the database in the presence of different light conditions and optic expansion information (Goyal et al., 2021a). During these landing maneuvers, bumblebees experienced three different levels of dim light intensities varying from twilight to sunrise and two visual patterns of the landing platform. These are a checkerboard pattern and a spoke pattern, which contain relatively high and low optical expansion information, respectively.

## Results

Our previous study on the landing dynamics of bumblebees showed that bumblebees hold the relative rate of expansion constant at a desired set-point only for brief time intervals. In between these intervals, bumblebees stepwise regulate this set-point as they reach closer to the landing platform (Goyal et al., 2021a). Here, we used the temporal dynamics of relative rate of expansion resulting from this stepwise regulation of set-points to study the underlying closed-loop sensorimotor control dynamics. For this, we used a newly developed algorithm to extract the track segments leading up to these brief constant-*r* segments (see STAR Methods). These segments are hereafter referred to as entry segments, as they potentially correspond to the moments when a bumblebee uses its sensorimotor control system to regulate relative rate of optical expansion and reach the desired set-point.

### Track segments leading to the constant-*r* segments are the transient phases of reaching the steady state set-point

By applying the entry segment detection algorithm to all landing trajectories recorded by Goyal et al. (2021a) and available in Goyal et al. (2021b), we identified 2,776 flight sections in which a constant-*r* segment was preceded by an entry segment. In each of the 2,776 combined pairs of entry segment and constant-*r* segment, we used the low-pass filtered temporal dynamics of relative rate of expansion (*r*_f_(*t*)) to identify first-order to third-order transfer functions that can explain the variation of *r*_f_(*t*) with *r*^∗^(*t*) as an input. Here, *r*^∗^(*t*) is a signal set constant at a desired set-point value. For the first-, second-, and third-order transfer functions, the fit percentages were *F*_1_ = 87.9% (84.3%–90.2%), *F*_2_ = 98.2% (94.6%–99.1%) and *F*_3_ = 98.8% (96.7%–99.5%), respectively (median [interquartile range]; see STAR Methods, Equation 2 for the definition of fit percentage *F*). Based on these percentages, we chose the second-order transfer functions to describe the observed *r*_f_(*t*) dynamics because the second-order transfer functions captured more variation than the first-order transfer functions, and a similar variation as third-order transfer functions.

**Figure 3:**
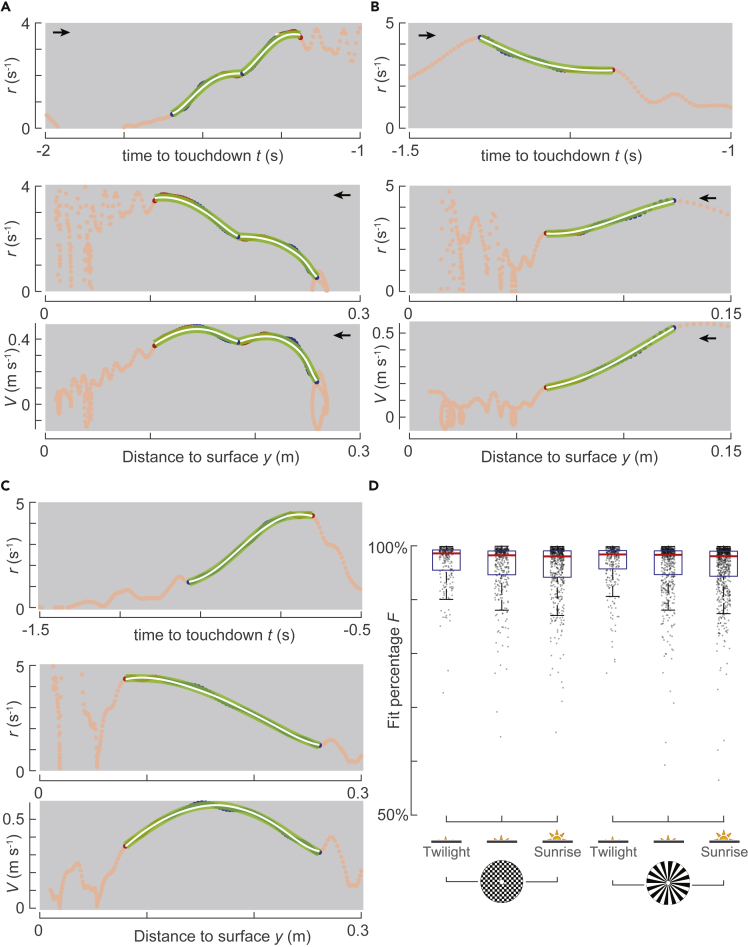
**The time-evolution of relative rate of expansion in a combined entry and constant-*r* segment can be captured by a single dynamic system** (A–C) Three examples of approach flights of bumblebees landing on a spoke landing platform in the sunrise light condition. The landings were initiated from free-flight (A, B) and after take-off (C), and in all landing maneuvers, the bumblebees reached the desired optical expansion set-point *r*^∗^ at least once (red). The top subpanel shows the variation of relative rate of expansion *r* with time to touchdown *t*, and the middle and bottom subpanels show the variation with distance to the landing surface *y* of relative rate of expansion *r* and approach velocity *V*, respectively. Each panel shows the complete track (orange), the constant-*r* segment (red), the transient entry segment (blue), the low-pass filtered signal used for system identification *r*_f_(*t*) (light green), and the estimated output from the simulation of identified second-order transfer functions *r*_s_(*t*) (white). The black arrow indicates the direction in which abscissa data varies as a bumblebee approaches the landing disc. (D) The goodness of the fit of the model simulation result *r*_s_(*t*) with the low-pass filtered signal *r*_f_(*t*), defined as the fit percentage of the normalized root-mean-square errors (Equation 2), for all six tested treatment conditions. For each condition, we show a boxplot and the fit percentage for pairs of entry and constant-*r* segments (dots). See also Figure S3.

The identified transfer functions captured the dynamics of relative rate of expansion in different cases, including when bumblebees were accelerating or decelerating during the entry segments (Figures 3A and 3B, respectively), or when they were doing both (Figure 3C). These transfer functions also captured the dynamics of relative rate of expansion during entry and constant-*r* segments when bumblebees landed in the presence of different landing patterns (checkerboard and spoke) and light conditions (twilight to sunrise) or when they landed from a free-flight or immediately after a take-off (Figure 3D).

For a pair of entry and constant-*r* segment, these results show that the low-pass filtered temporal dynamics of relative rate of expansion in the entry segment can be described by a second-order dynamic process that is aiming to reach the steady-state set-point of the relative rate of optical expansion. Hence, entry and constant-*r* segments contain transient and steady-state responses of the closed-loop sensorimotor control system of bumblebees, respectively; this suggests that bumblebees use relative rate of optical expansion *r* as a controlled variable in their sensorimotor control loop during the complete landing approach, including not only the constant-*r* segments but also the entry segments.

### Bumblebees modulate the transient response of their sensorimotor control system during their landing approach

To model the transient response of the sensorimotor control system of landing bumblebees, we estimated the expansion-acceleration $\dot{r_{\text{e}}}$ in each entry segment (see STAR Methods). It is a measure of how fast the sensorimotor control system of a bumblebee allows the animal to reaching the set-point. In 2,651 out of 2,776 entry segments, bumblebees started with a relative rate of expansion lower than their set-point and therefore, exhibited positive expansion-acceleration. In the remaining 125 entry segments, bumblebees started with a higher relative rate of expansion than their set-point and thus exhibited negative expansion-acceleration to converge to the set-point. Because bumblebees mostly increased their relative rate of expansion (during 95% of all identified cases), we focused on these positive expansion-acceleration entry segments in further analyses.

**Figure 4:**
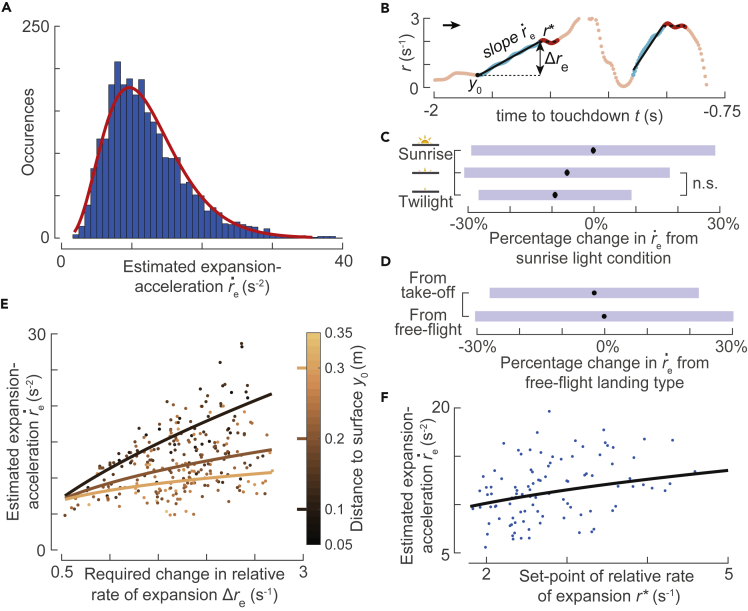
**Bumblebees modulate the optical expansion-acceleration during the transient phase of their landing maneuvers with environmental conditions** (A) Histogram of estimated expansion-acceleration $\dot{r_{\text{e}}}$ during all transient phases in which bumblebees increased their expansion rate (n = 2,651 segments, red curve indicates the fitted gamma distribution). (B) An example track depicting the flight parameters that define the kinematics of a transient segment when converging to the optic expansion set-point *r*^∗^; we use these flight parameters for our linear mixed model analyses. (B–F) In our model, we correlated the change in the expansion-acceleration ($\dot{r_{\text{e}}}$) during entry segments with five covariates: light intensity; landing type (landing from take-off or free flight); set-point of relative rate of expansion *r*^∗^; required step change in relative rate of expansion Δ*r*_e_; the starting distance of the entry segment *y*_0_). See Table 1 for statistical model output. (C and D) The effect of light conditions (C) and landing type (D) on the expansion-acceleration ($\dot{r_{\text{e}}}$) during the transient phase of the landing maneuvers of bumblebees, as determined by our linear mixed model. Black dots depict the estimated means, blue bars are 95% confidence intervals, and nonsignificant differences are indicated on the right. (C) On average, bumblebees flying in the sunrise light condition converge to the desired set-points at significantly higher expansion-accelerations than in the other lower light intensities. (D) Equivalently, bumblebees landing directly after take-off reach their set-points at slightly lower expansion-accelerations than when landings were initiated from free-flight. (E and F) The effect of the landing condition parameter set (*r*^∗^, Δ*r*_e_, *y*_0_) on the expansion-acceleration ($\dot{r_{\text{e}}}$) during the transient phase of the landing maneuvers of bumblebees, as determined by our linear mixed model. Here, we show the model results for landings initiated from free-flight in the sunrise light condition; see Figures S5 and S6 for landings that follow take-off and for other light conditions. (E) The effect of Δ*r*_e_ and *y*_0_ on $\dot{r_{\text{e}}}$, showing that bumblebees converge to *r*^∗^ at higher expansion-accelerations when flying closer to the landing platform (lower *y*_0_) and when they need to achieve a larger change in optical expansion value (larger Δ*r*_e_). Curves depict the statistical model output for the median value *r*^∗^ = 2.78 s^−1^, and data points are shown for the interval $r^{*}\in \left[2.28,\;3.28\right]$ s^−1^ centered around the median value. (F) The expansion-acceleration $\dot{r_{\text{e}}}$ increases with the set-point value *r*^∗^, independent of other conditional variables (no significant interaction). This shows that bumblebees converge more rapidly (larger $\dot{r_{\text{e}}}$) to higher set-point *r*^∗^ than to lower values of *r*^∗^. The curve shows the model prediction of $\dot{r_{\text{e}}}$ versus *r*^∗^ at the median values of Δ*r*_e_ and *y*_0_; data points are plotted for the intervals $\text{Δ}r_{\text{e}}\in \left[1.28,\;2.08\right]$ s^−1^ and $y_{0}\in \left[0.18,\;0.24\right]$ m centered around their median values. See also Figures S1, S2, S5, S6, and S10.

The estimated optical-expansion-acceleration ($\dot{r_{\text{e}}}$) varied considerably among entry segments (Figure 4A). The observed distribution of $\dot{r_{\text{e}}}$ can be approximated by the gamma distribution (median $\dot{r_{\text{e}}}$ = 11.07 s^−2^, *a* = 4.8 [4.5, 5.0], *b* = 2.5 [2.4, 2.7], mean [95% confidence intervals]) (Evans et al., 2000).

We further used a linear mixed-effects model to test how the observed optical-expansion-acceleration ($\dot{r_{\text{e}}}$) varied with the initial distance from the landing surface (*y*_0_), the step change required in the relative rate of expansion during an entry segment (Δ*r*_e_), and the set-point of relative rate of expansion that a bumblebee aims to reach at the end of an entry segment (*r*^∗^) (Figure 4B; STAR Methods). We found linear variation between the logarithmic transformations of *y*_0_, Δ*r*_e_ and *r*^∗^ with $\dot{r_{\text{e}}}$ (Table 1). Moreover, we found that there were no significant interactions among these covariates except between *y*_0_ and Δ*r*_e_. Therefore, we first describe the effects of *y*_0_ and Δ*r*_e_ together and then the effects of *r*^∗^.

**Table 1:** Linear mixed-effects model results of how bumblebees modulate the expansion-acceleration during entry segments

Fixed effect	estimate	SE	t-value	p value
α (twilight, landing from free-flight)	1.35	0.05	28.24	<0.001
β_1_ (log(*y*_0_))	−0.29	0.02	−11.57	<0.001
β_2_ (medium light)	0.03	0.02	2.07	0.039
β_3_ (sunrise)	0.10	0.01	6.55	<0.001
β_4_ (landing after take-off)	−0.02	0.01	−1.99	0.047
β_5_ (log(Δ*r*_e_))	−0.16	0.05	−3.01	0.003
β_6_ (log(*r∗*))	0.32	0.03	12.48	<0.001
β_7_ (log(*y*_0_) × log(Δ*r*_e_))	−0.36	0.03	−11.17	<0.001

The dependent parameter is the expansion-acceleration during the entry segment ($\dot{r_{\text{e}}}$), and independent parameters are the starting distance from the landing surface (*y*_0_), the required step-change in relative rate of expansion (Δ*r*_e_), the final set-point to reach (*r*^∗^), light condition, and the landing type (from free-flight or after take-off). The data comprise 2,651 entry segments identified in 2,511 landing maneuvers, and the statistical model is defined in Equation 7. For details, see STAR Methods and Figures 4, S5, and S6.

The linear mixed-effects model analysis shows that bumblebees reached their set-points at a higher rate, i.e. they exhibited higher optical-expansion-acceleration $\dot{r_{\text{e}}}$ when they were closer to the landing surface (lower *y*_0_) and when their set-point *r*^∗^ was further away from their initial relative rate of expansion *r*_0_ (higher Δ*r*_e_ = *r*^∗^– *r*_0_) (Table 1). However, due to the presence of significant interaction between *y*_0_ and Δ*r*_e_, the differences in $\dot{r_{\text{e}}}$ due to these trends depend on the actual values of *y*_0_ and Δ*r*_e_ (Figure 4E). The bumblebees increased their expansion-acceleration $\dot{r_{\text{e}}}$ with the required step-change Δ*r*_e_ at a higher rate when they were closer to the landing surface, than when they were further away from it. In an average landing maneuver, a bumblebee reached its set-point at a 78% higher rate when they were 0.1 m from the landing surface than at *y*_0_ = 0.3 m ($\dot{r_{\text{e}}}$ = 17.1 [0.5] s^−2^ and $\dot{r_{\text{e}}}$ = 9.6 [0.3] s^−2^, respectively) (mean [SE]). Similarly, on average, bumblebees exhibited 31% higher expansion-acceleration when their set-point was twice as far from their initial set-point ($\dot{r_{\text{e}}}$ = 11.6 [0.3] s^−2^ and $\dot{r_{\text{e}}}$ = 8.9 [0.3] s^−2^, for Δ*r*_e_ = 2 s^−1^ and Δ*r*_e_ = 1 s^−1^, respectively). The model also predicted that bumblebees approach their set-point at a higher rate ($\dot{r_{\text{e}}}$) for higher set-points (p value < 0.001, Table 1) (Figure 4F). On average, the bumblebees exhibited 14% higher expansion acceleration when *r*^∗^ = 3 s^−1^ as compared with *r*^∗^ = 2 s^−1^ ($\dot{r_{\text{e}}}$ = 11.3 [0.3] s^−2^ and $\dot{r_{\text{e}}}$ = 10.0 [0.3] s^−2^, respectively).

These results show that the sensorimotor control system of bumblebees responds faster when (a) they are closer to the surface, (b) they have a higher goal (set-point), and (c) their goal is further away from the relative rate of expansion at the start of the entry segment.

### Bumblebees exhibit slower sensorimotor control response in lower light intensities

The aforementioned linear mixed-effects model also allowed us to predict how bumblebees adapt the response of their sensorimotor control system with changes in the environmental conditions (expansion information and light intensity). The model shows that the closed-loop transient response of bumblebees, expressed as expansion-acceleration $\dot{r_{\text{e}}}$, did not significantly differ between landings on a platform with relatively high and low optic expansion information (i.e. the platforms with checkerboard and spokes pattern, respectively; p value = 0.19). It implies that the sensorimotor control system of bumblebees is robust to the tested variation in expansion information and exhibits similar performance in the presence of both landing patterns.

In contrast to the effect of landing patterns, the decrease in light intensity diminishes the transient response characteristic of the vision-based sensorimotor control system of bumblebees. On average, the bumblebees reached their set-point at a 9% lower expansion acceleration in twilight conditions than in sunrise (Figure 4C). This reduction in $\dot{r_{\text{e}}}$ with light intensity holds for all values of *r*^∗^, *y*_0_, and Δ*r*_e_ (Table 1). This shows that, starting from an initial value of relative rate of expansion, bumblebees take more time to reach a set-point in lower light intensity as compared with a higher light intensity.

### Bumblebees landing after take-off reach their set-points slightly slower than bumblebees landing from free-flight

Using the same linear mixed-effects model, we also tested how the rate at which the bumblebees reached their set-points differed between the landings from free-flight and the landings that were performed directly after taking off. The model shows that the bumblebees reached their set-point only at a 3% lower optic expansion acceleration ($\dot{r_{\text{e}}}$) when they landed after take-off than when they landed from free-flight (Figure 4D); this also holds for all values of *r*^∗^, *y*_0_, and Δ*r*_e_ (p value = 0.047, Table 1); this shows that the sensorimotor control response of bumblebees depends to a small amount on how a bumblebee initiates its landing.

### Bumblebees use the transient response of their sensorimotor control system to mostly accelerate toward the landing surface

**Figure 5:**
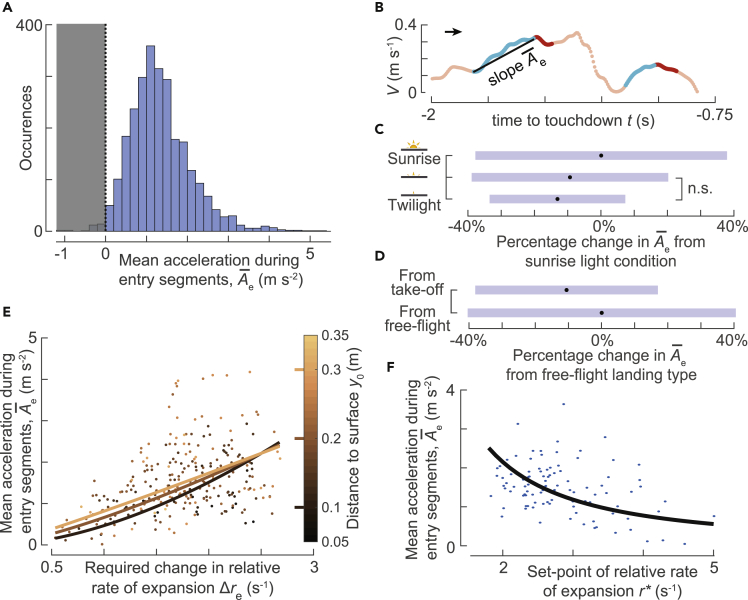
**To reach their required optical expansion set-point, bumblebees robustly accelerate toward the landing surface during the transient phases of their landing maneuver** (A) Histogram of mean acceleration of the bumblebee toward the landing platforms (${\bar{A}}_{\text{e}}$) during all identified entry segments (n = 2651 segments). During 99% of the entry segments, bumblebees accelerated toward the platform (${\bar{A}}_{\text{e}}$ > 0) and in only 1% they decelerated during the transient flight phase. (B) An example track depicting the mean acceleration of the bumblebee ${\bar{A}}_{\text{e}}$ during the entry segment; positive values thus depict an acceleration toward the landing platform. (C–F) The results of our linear mixed model in which we correlated the mean acceleration of the bumblebee ${\bar{A}}_{\text{e}}$ during the entry segments with five conditional parameters: light intensity; landing type (landing from take-off or free flight); the set-point of relative rate of expansion *r*^∗^; the required step change in relative rate of expansion Δ*r*_e_; the starting distance of the entry segment *y*_0_. See Table 2 for statistical model output. (C and D) The effect of light intensity (C) and landing type (D) on the acceleration of the bumblebee ${\bar{A}}_{\text{e}}$ during the entry segments. Black dots depict the estimated means, blue bars are 95% confidence intervals, and nonsignificant differences are indicated on the right. (C) On average, bumblebees flying in the sunrise light condition accelerated faster toward the landing platform during the transient phase than in the lower light intensities. (D) Bumblebees also accelerated faster toward the platform when they landed from a free-flight condition than following a take-off. (E and F) The effect of the landing condition parameter set (*r*^∗^, Δ*r*_e_, *y*_0_) on the acceleration of the bumblebee ${\bar{A}}_{\text{e}}$ during the entry segments, as determined by our linear mixed model. Results are shown for landings initiated from free-flight and in the sunrise light condition; see Figures S7 and S8 for the equivalent results for landings that follow take-off and for other light conditions. (E) The effect of change required in the expansion rate (Δ*r*_e_) and distance from the platform (*y*_0_) on body acceleration ${\bar{A}}_{\text{e}}$. The curves depict the statistical model output at the median value *r*^∗^ = 2.78 s^−1^, and data points are shown for the interval $r^{*}\in \left[2.28,\;3.28\right]$ s^−1^. The model shows that the body acceleration is higher when the bumblebee needs to achieve a larger change in optical expansion value (larger Δ*r*_e_); although significant, body accelerations vary only little with distance from the platform (*y*_0_). (F) Body accelerations toward the platform ${\bar{A}}_{\text{e}}$ decrease with an increase in the relative rate of expansion set-point *r*^∗^, independent of all other parameters. The curve shows the model prediction of ${\bar{A}}_{\text{e}}$ versus *r*^∗^ at the median values of Δ*r*_e_ and *y*_0_; data points are plotted for the intervals $\text{Δ}r_{e}\in \left[1.28,\;2.08\right]$ s^−1^ and $y_{0}\in \left[0.18,\;0.24\right]$ m around these median values. See also Figures S1, S4, S7, and S8.

To study how the sensorimotor control response affects the flight dynamics, we further looked at the average acceleration ${\bar{A}}_{\text{e}}$ that bumblebees exhibited during these entry segments (Figure 5; STAR Methods). The average acceleration provides information about how bumblebees modulated their motor control. Note that the positive average acceleration $\left({\bar{A}}_{\text{e}}>0\right)$ corresponds to bumblebees only accelerating toward the platform (Figure 2D) or mostly accelerating during their entry segments (Figure 2F). Negative average accelerations $\left({\bar{A}}_{e}<0\right)$ correspond to bumblebees decelerating when moving toward the platform.

As expected, in the 125 entry segments with negative Δ*r*_e_, bumblebees always decelerated while flying toward the surface $\left({\bar{A}}_{e}<0\right)$ to decrease their relative rate of expansion and reach the desired set-point. In the 2,651 entry segments with positive Δ*r*_e_, bumblebees mostly accelerated $\left({\bar{A}}_{\text{e}}>0\right)$ toward the landing surface (2,620 times) and few times slowly decelerated (31 times) while increasing their relative rate of expansion. Hence, it can be concluded that bumblebees use the transient response of their sensorimotor control system to mostly (94.4%) accelerate toward the landing surface.

We further tested how the positive average acceleration ${\bar{A}}_{\text{e}}$ varied with the relative rate of expansion set-point (*r*^∗^), the distance to the landing surface (*y*_0_), and the step change required in the relative rate of expansion during the entry segment (Δ*r*_e_) (Figure 5B; STAR Methods). The linear mixed-effects model shows that, on average, the bumblebees exhibited higher accelerations for higher step change Δ*r*_e_ and at a distance *y*_0_ further away from the landing surface (Table 2). But there is a significant interaction between *y*_0_ and Δ*r*_e_, and thus these trends depend on the actual values of *y*_0_ and Δ*r*_e_ (Figure 5E). On average, the landing bumblebees exhibited only 14% lower average acceleration at 0.1 m from the landing platform than at *y*_0_ = 0.3 m (${\bar{A}}_{e}=1.07\;\left[0.05\right]\;{\mathrm{ms}}^{-2}$ and ${\bar{A}}_{\text{e}}=1.25\;\left[0.05\right]\;{\mathrm{ms}}^{-2}\text{,}$ respectively). In contrast with *y*_0_, bumblebees showed stronger variation in average acceleration with the required step change Δ*r*_e_ in an entry segment. On average, bumblebees exhibited a 142% higher average acceleration when the required step change is doubled from Δ*r*_e_ = 1 s^−1^ to Δ*r*_e_ = 2 s^−1^ (${\bar{A}}_{\text{e}}=0.51\;\left[0.02\right]\;ms^{-2}$ and ${\bar{A}}_{\text{e}}=1.23\;\left[0.05\right]\;ms^{-2}$, respectively). This shows that bumblebees accelerate toward the landing surface, albeit with smaller acceleration, even when they are close to the landing surface. Moreover, the average acceleration with which bumblebees advance toward the landing surface is larger when the required relative rate of expansion step change is also large.

**Table 2:** Linear mixed-effect model results of how bumblebees modulate their acceleration toward the landing surface during entry segments

Fixed effect	Estimate	SE	t-value	p value
α (twilight, landing from free-flight)	1.77	0.08	21.42	<0.001
β_1_ (log(*y*_0_))	0.51	0.05	11.17	<0.001
β_2_ (medium light)	0.04	0.03	1.54	0.123
β_3_ (sunrise)	0.14	0.03	5.28	<0.001
β_4_ (landing after take-off)	−0.11	0.02	−5.12	<0.001
β_5_ (log(Δ*r*_e_))	0.42	0.10	4.20	<0.001
β_6_ (log(*r∗*))	−1.47	0.05	−30.63	<0.001
β_7_ (log(*y*_0_) × log(Δ*r*_e_))	−0.55	0.06	−9.40	<0.001

The dependent parameter is the average acceleration during the entry segment (${\bar{A}}_{\text{e}}$), and independent parameters are the starting distance from the landing surface (*y*_0_), the required step-change in relative rate of expansion (Δ*r*_e_), the final set-point to reach (*r*^∗^), light conditions, and the landing type (from free-flight or after take-off). The data comprise 2,620 entry segments identified in 2,485 landing maneuvers with positive average acceleration (${\bar{A}}_{\text{e}}>0$). The statistical model is defined as Equation 7. For details, see STAR Methods and Figures 5, S7, and S8.

The linear mixed-effects model for ${\bar{A}}_{\text{e}}$ also predicted that the bumblebees exhibited a decrease in the average acceleration with an increase in set-point *r*^∗^ (p value < 0.001, Table 2 and Figure 5F). On average, landing bumblebee exhibited a 45% lower average acceleration when *r*^∗^ = 3 s^−1^ than when *r*^∗^ = 2 s^−1^ (${\bar{A}}_{\text{e}}=1.26\;\left[0.05\right]\;{\mathrm{ms}}^{-2}$ and ${\bar{A}}_{\text{e}}=2.29\;\left[0.1\right]\;{\mathrm{ms}}^{-2}$, respectively). This decrease in ${\bar{A}}_{\text{e}}$ with increasing set-point is observed because bumblebees decelerate more in an entry segment with a higher set-point.

Similar to the expansion-acceleration $\dot{r_{e}}$, we also assessed how the level of the radial expansion of optic flow, light intensity, or landing type affected the mean acceleration ${\bar{A}}_{\text{e}}$ during an entry segment. We found that bumblebees accelerated during entry segments in a similar manner in the presence of both strong and weak expansion information (checkerboard and spoke pattern, respectively). Moreover, bumblebees accelerated 13% less rapidly in twilight as compared with the sunrise light condition (Figure 5C). In addition, bumblebees exhibited 10% lower accelerations when they landed directly after a take-off than from a free-flight condition (Figure 5D). These results hold for all *r*^∗^, *y*_0_, and Δ*r*_e_ (Table 2). This shows that bumblebees use the transient response of their sensorimotor control system to accelerate toward the landing surface in the presence of different levels of optic expansion information, light conditions, and when they land after a take-off or directly from a free-flight.

## Discussion

Here, we studied the sensorimotor control dynamics of landing bumblebees and how these bumblebees use their control system to advance toward a vertical landing surface. For this purpose, we used 10,005 previously recorded landing maneuvers of bumblebees (Goyal et al., 2021b). These landings resemble those produced by bumblebees foraging in nature, i.e. when they fly from one flower to another within a flower patch or between flower patches and their hive (Heinrich, 1979; Goyal et al., 2021a). We also find how landing bumblebees adapt their sensorimotor control response when they land after a take-off or from free-flight condition, in the presence of different levels of optic expansion information and at various dim light intensities.

### Bumblebees use their sensorimotor control system during landing to regulate relative rate of optical expansion and produce motor actions to reach a desired set-point

During their landing approach, bumblebees tend to fly at a specific set-point of relative rate of expansion for only short periods of time, and they regularly switch to a new set-point (Goyal et al., 2021a). When they regulate the relative rate of expansion during landing, the stepwise modulation in the set-points means that bumblebees not only fly at the set-point but must also exhibit a transient entry phase to reach their set-point—a typical attribute of a step-response (Ogata, 2010). Moreover, during these transient phases, bumblebees should produce motor output to bring their relative rate of expansion closer to the desired set-point. We used the natural stepwise excitation of this dynamic system to parse the underlying sensorimotor control system of landing bumblebees.

Doing so, we showed that the time evolution of relative rate of expansion, both in entry and constant-*r* segments, can be captured by a single dynamic system (Figure 3). We also show that bumblebees tended to decelerate during an entry segment when they had to reduce their relative rate of expansion and mostly accelerated to increase it. This means that bumblebees produce motor output in a direction that is consistent with the requirement of increasing or decreasing their relative rate of expansion in an entry segment. Moreover, our results show that bumblebees exhibited higher mean acceleration in an entry segment when they started with a relative rate of expansion further away from their set-point (i.e., higher Δ*r*_e_) (Figure 5E); this shows that the motor output produced by bumblebees is dependent on the step change required in an entry segment.

We also performed an additional analysis to check how the instantaneous motor output produced by bumblebees in an entry segment is correlated with the difference between the desired set-point and the instantaneous relative rate of expansion (Δ*r*_e_). For this purpose, we computed the mean acceleration vector *A*_e_(*t*) by simulating a constant expansion-acceleration motion in an entry segment. We did this because the acceleration vector *A*(*t*) as obtained from double differentiation of position vector was noisy. We found a high and statistically significant correlation between *A*_e_(*t*) and Δ*r*(*t*) for all 2,776 entry segments (correlation coefficient: 0.988 [0.976 0.994], median [interquartile range], all p values < 0.001).

Considered together, all these results show that the sensorimotor control system of landing bumblebees regulates the relative rate of expansion to advance toward the landing surface, i.e., they produce motor output based on the difference between the instantaneous relative rate of expansion and the current set-point.

Using a system identification approach, we found that the second order transfer functions adequately captured the dynamics of bumblebees in both entry and constant-*r* segments; this is similar to classical mechanics where motions can be captured using second-order derivatives. Second-order models are also found to be adequate for modeling the lift response in tethered flying bumblebees (Tanaka and Kawachi, 2006).

The median values of parameters identified from system identification are close to each other (Figure S3), but we could not use the identified model order or its parameters to test how bumblebees adjust their sensorimotor control in different conditions; this is because several sets of parameter combinations can approximate the temporal dynamics of *r* during a combined pair of entry and constant-*r* segments, and the line search algorithms used during optimization could find these parameter combinations as local minima. Therefore, we used system identification only to capture the approximate (low-frequency) dynamics and provide evidence that the dynamics of *r* during entry segments is the response of sensorimotor control system when a bumblebee is away from its steady-state. For understanding how bumblebees modulate their transient response, we use another method where we approximate their transient response as a motion at a constant expansion-acceleration $\dot{r_{\text{e}}}$.

### How do bumblebees modulate the transient response of their sensorimotor control system during landing?

During entry segments, we found that the transient response of the sensorimotor control system of bumblebees can be modeled as a motion at a constant expansion acceleration $\dot{r_{\text{e}}}$ (Figure S2). This response is similar to the classic attribute of step response of linear controllers (Ljung, 1999; Ogata, 2010) that are identified in animal flight (for review, see Taylor et al., 2008; Cowan et al., 2014; Roth et al., 2014; Dickinson and Muijres, 2016 and for examples of step-response in flight, see Baird et al., 2005; Fry et al., 2009; Rohrseitz and Fry, 2011; Fuller et al., 2014).

We further tested the dependency of the response of sensorimotor control ($\dot{r_{\text{e}}}$) on the three other state parameters: the relative rate of expansion set-point (*r*^∗^), the starting distance from the landing platform (*y*_0_), and the required step-change in relative rate of expansion (Δ*r*_e_) (Figure 4). We found that bumblebees approached their goal at a higher expansion-acceleration when they were closer to the platform and when both their goal and step-change were higher. This modulation of the transient response enables bumblebees to reach their set-point rapidly and robustly, especially when close to the platform.

The modulation of the transient response can be advantageous for bumblebees, as flying at a set-point ensures deceleration and can provide the animal an estimate of the distance to the platform based on which a change in the set-point could be triggered (Croon and De Croon, 2016; Goyal et al., 2021a).

The observed modulation in response rate $\dot{r_{\text{e}}}$ with aforementioned parameters can occur due to two possible reasons. First, it can be due to the active adjustment of the underlying dynamic system, e.g., by changing the controller gains with distance to the platform to delay the occurrence of instabilities (Croon and De Croon, 2016). Physiologically, changes in controller gains would translate into changes in wingbeat and body kinematics in such a way that higher aerodynamic forces are produced in case of higher $\dot{r_{\text{e}}}$ at a given set-point and instantaneous relative rate of optical expansion. Second, it can be an inherent characteristic of a closed-loop controller with relative rate of expansion as a controlled variable, such as the increase in the transient slope with the set-point (Ogata, 2010) or with decreasing proximity to the landing surface (Corke and Good, 1992). A new study, possibly involving the open-loop characterization of subsystems (sensory system, controller and motor system), is needed to identify the contribution of each cause to the observed response modulation.

### The sensorimotor control system of bumblebees compensates for low-light conditions by responding more slowly

We also tested how the change in levels of optic expansion information available from the landing platform and light intensity affected the closed-loop sensorimotor response of bumblebees. We found that the transient response of bumblebees is robust to the tested variation in expansion information. However, bumblebees exhibited on average a 9% lower expansion-acceleration in twilight than in sunrise light condition; this shows that in the lowest dim light condition, bumblebees not only exhibit lower set-points (Goyal et al., 2021a) but also the slope at which they reach this set-point is lower. These results are in agreement with the findings from literature where animals, including bumblebees, are shown to fly more slowly in low light intensity due to a loss in the temporal resolution of the visual system (Rose and Menzel, 1981; Spiewok and Schmolz, 2006; Reber et al., 2015, 2016b; Sponberg et al., 2015).

In principle, the diminishing transient response with light intensity can occur due to two reasons. First, it can be due to a decrease in the response speed of photoreceptors (Reber et al., 2015) and a consequent expected increase in the latency of relative rate of expansion measurement in low light intensity. Second, it can be due to the active adjustment of the closed-loop dynamic system by bumblebees, as vision becomes less reliable in low light intensity. Irrespective of the cause, our results show that the diminished response in lower light intensity leads to bumblebees exhibiting lower average accelerations toward the landing surface (Figure 5C); this signifies that bumblebees use a more cautious landing approach in lower light intensities.

### Landing bumblebees use (1) the steady-state response of their sensorimotor control system to always decelerate and (2) the transient response to mostly accelerate toward the landing surface

We analyzed how the transient response observed during entry segments influenced the average acceleration ${\bar{A}}_{\text{e}}$ during entry segments (Figure 5). We did this because these accelerations result from changes in the aerodynamic forces produced by the motor response (wingbeat and body kinematics), which should depend on the difference between the instantaneous relative rate of expansion and the set-point.

The linear mixed-effects model on acceleration ${\bar{A}}_{\text{e}}$ confirms this dependence, as it predicts that bumblebees exhibited a 142% higher average acceleration when the required step change is doubled. Next to this, ${\bar{A}}_{\text{e}}$ also increased with *y*_0_, and light intensity, but it decreases with increasing set-point *r*^∗^. The average acceleration is also lower when landing after a take-off than from a free-flight; this is because bumblebees when landing from take-off more often exhibited a transient phase in which they decelerated for a brief period after accelerating (Figure S4). This deceleration during entry segments results in lower mean accelerations.

On average, bumblebees mostly (99%) accelerated toward the landing surface as they increased their instantaneous relative rate of optical expansion to reach their next set-point. They accelerated toward the surface during entry segments when they landed after a take-off or from a free-flight and in the presence of different levels of optic expansion information and light intensities. They even accelerated when they were close to the landing surface, after which they rapidly decelerated to achieve a gentile touchdown. These results help us understand how foraging bumblebees visit flowers at very high rates of up to 1000 visits per hour (Heinrich, 1979). Moreover, the acceleration phases during landing are also found in other bees (Shackleton et al., 2019; Tichit et al., 2020a, 2020b) and flies (Liu et al., 2019), making it likely that it is a generic feature of the landing strategy of flying insects.

In our previous study, we showed that bumblebees decelerate during constant-*r* segments (Goyal et al., 2021a). In this study, we show that bumblebees accelerated toward the landing surface during the entry segments in between the constant-*r* segments. Moreover, the entry and constant-*r* segments are the transient and steady-state response of a system aiming to reach its desired optic expansion set-point value. Considered together, these results show that bumblebees use the transient and steady-state response of their optical-expansion-rate-based sensorimotor control system to accelerate and decelerate, respectively, as they advance toward the landing surface.

### How do bumblebees perform their flight control during landing?

What actions could bumblebees perform during flight to produce accelerations (or decelerations) for reaching their set-points? Flies regulate their ground speed by using the translatory optic flow as sensory input, and body tilt as their motor output. This tilt of the body reorients their aerodynamic thrust vector, making the animal accelerate or decelerate forward (David, 1978; Rohrseitz and Fry, 2011; Medici and Fry, 2012). For bumblebees, the pitch angle of their body is associated with determining the thrust angle and forward flight speed (Dudley and Ellington, 1990). Therefore, similar to flies, bumblebees could also use the pitch angle of their body as a high-level control to mediate acceleration during landing in a nested loop architecture (Medici and Fry, 2012). More detailed measurements including an estimation of body orientation and possibly wingbeat kinematics would be needed to test this hypothesis.

### Conclusion

In this study, we investigated how bumblebees land using a modular landing strategy. We analyze the closed-loop response of their sensorimotor control system that parses visual information. We found that bumblebees use such a system to reach and fly at their desired set-point of relative rate of optical expansion. Such a system offers a way of both accelerating and decelerating toward the landing surface robustly in different environmental conditions and can thus be considered as a functional mechanism that bumblebees possess to perform rapid landings. Similar acceleration and deceleration phases have been observed in landings of other insects, suggesting that the here-described landing strategy might be generic to flying insects. Moreover, the here-proposed landing control system can be used as bio-inspiration for landing control systems on board of man-made flying machines (Shyy et al., 2016; Karásek et al., 2018). An onboard controller that uses the relative rate of optical expansion as a controlled variable (Croon and De Croon, 2016; Ho et al., 2017) can be modified to use the stepwise modulation of relative rate of optical expansion, which would lead to rapid landings.

### Limitations of the study

The set-point detection algorithm used in this study does not capture all of the set-points that bumblebees exhibit during landing (Goyal et al., 2021a). It misses the set-points at which bumblebees fly for some time period, but the variation in *r* in the corresponding flight segments is higher than the threshold dictated by scale factor *f*. In addition, it also misses the set-points that a bumblebee does not reach during its approach flight; this happens for example when a bumblebee changes to a new set-point before it reaches the current set-point or when it lands or aborts the landing before reaching its set-point. Similarly, the algorithm used in this study to extract the entry segments (Methods S1 in the Data and code availability) only captures the track segments that have at least one constant-*r* segment and an associated monotonous variation of *r* preceding the constant-*r* segments. Despite these limitations, the response adjustment of the control system during entry segments and its adjustment with environmental conditions can be accurately captured by analyzing many landing maneuvers, as we did in this study.

## STAR★Methods

### Key resources table

**Table:** 

REAGENT or RESOURCE	SOURCE	IDENTIFIER
**Deposited data**		

Landing manoeuvres of bumblebees	Goyal et al., 2021a, Goyal et al., 2021b	Mendeley Data: https://doi.org/10.17632/rrbjyhkm8z.1

**Experimental models: Organisms/strains**		

*Bombus terristris*	Koppert B.V.	Koppert strain

**Software and algorithms**		

Methods S1	This paper	https://github.com/kaku289/nimble-bbee-analysis/tree/entryTransients
Methods	Goyal et al., 2021a	https://github.com/kaku289/nimble-bbee-analysis/tree/rref
Matlab R2020a	Mathworks Inc	https://www.mathworks.com/
R 4.0.2	R Foundation	https://cran.r-project.org/bin/windows/base/old/4.0.2/

### Resource availability

#### Lead contact

Further information and requests for resources should be directed to and will be fulfilled by the lead contact, Florian T. Muijres (florian.muijres@wur.nl).

#### Materials availability

This study did not generate new unique reagents.

### Experimental model and subject details

This study reports on the landing dynamics of bumblebees (*Bombus terristris*) from a commercially available bumblebee colony provided by Koppert B.V. (Berkel en Rodenrijs, The Netherlands). As food source, the colony had *ad libitum* access to pollen and a 50% sugar solution. During the experiments, the pollen was available in the hive, whereas the 50% sugar solution was provided on the opposite side of the flight arena (see below for details).

### Method details

In this study, we analyzed the landing maneuvers of bumblebees to elucidate the dynamic characteristics of the closed-loop sensorimotor control system that bumblebees use during landing. The dataset of landing maneuvers that we used for this analysis is available at Goyal et al. (2021b). The in-depth details of the experimental set-up and the estimation of relevant state variables that were used to capture these landing maneuvers is provided in Goyal et al. (2021a); therefore, we here present these aspects succinctly. The methodological aspects new to this study are explained in detail.

#### Experimental set-up

The experimental setup consisted of a bumblebee hive, a 50% sugar-solution food-source, a flight arena and a real-time machine-vision based videography system (Figures 2A and 2B). The hive and food-source were placed opposite to each other with a flight arena (3 × 0.48 × 0.48 m; length×width×height) in between. Each of them was connected to a transparent Plexiglass tube that extended 0.07 m inside the flight arena and had a vertical landing disc (0.18 m diameter) attached at the end. These landing platforms were covered with either a black-and-white checkerboard pattern (0.01 m squares) or a black-and-white spoke pattern (32 spokes).

The flight arena was illuminated by a white broad-spectrum LED light panel that was set to provide three different levels of environmental light intensity: a light condition resembling twilight (light intensity = 13.7 lx, irradiance = 0.041 W m^−2^, radiance = 0.011 W m^−2^ sr^−1^), a light condition resembling sunrise (144.9 lx, 0.440 W m^−2^, 0.118 W m^−2^ sr^−1^), and an intermediate light condition (33.3 lx, 0.1 W m^−2^, 0.027 W m^−2^ sr^−1^). These light conditions were measured using a spectroradiometer (Specbos 1211 with JETI Lival software), positioned at both the center of the flight arena and at the landing platforms (Goyal et al., 2021a). The corresponding spectral light intensity distributions at all conditions and measured locations are shown in Figure S9. As bumblebees can forage in daylight conditions of up to 128,000 lx, all tested light conditions are regarded as dim light, and thus we will also refer to these as such (Reber et al., 2015, 2016b; de Vries et al., 2020).

During the experiments, the landing pattern was changed every day and bumblebees were exposed to all three light conditions twice a day, following a pseudo-random treatment schedule (see Goyal et al., 2021a for details) (Figures 2A and 2B).

The landing maneuvers of bumblebees in the flight arena were tracked using a real-time machine-vision based videography system that included four synchronized cameras operating at 175 frames per second. The recorded maneuvers corresponded to bumblebees that were initially flying freely in the arena and then landed on a platform, or bumblebees that just took off either from the ground or the opposite platform, and subsequently landed on the other platform. These two types of landing were referred to as landings from free-flight and landings directly after take-off (Goyal et al., 2021a). Note that landings performed directly after take-off from the same landing platform were excluded from the analysis, because these might be uncontrolled landings following a failed landing attempt.

Each landing maneuver was expressed in a Cartesian coordinate system (Figure 2B) which has its origin at the center of the landing platform, *y*-axis normal to the platform and *z*-axis vertically upwards. To reduce tracking noise, landing maneuvers were filtered using a low-pass second-order two-directional Butterworth filter (*filtfilt* in Matlab 2020a) with a cut-off frequency of 20 Hz. We then stored these maneuvers as space-time arrays **X** = (*x,y,z,t*) with time *t* set to zero when the bumblebee reached the closest distance to the platform. We also computed the corresponding velocity and acceleration vectors (**U** = (*u,v,w*) and **A**=(*a*_*x*_*,a*_*y*_*,a*_*z*_), respectively) by numerically differentiating the space-time arrays **X** using a second-order central differencing scheme.

#### Estimation of state variables of the landing bumblebee

To analyze the landing dynamics of bumblebees, we first computed for each maneuver the temporal dynamics of four state variables: normal distance from the platform *y*(*t*), flight velocity towards the platform *V*(*t*)*=−v*(*t*), acceleration towards the platform *A*(*t*) = −*a*_*y*_(*t*), and the instantaneous relative rate of expansion that a bumblebee experienced due to its motion normal to the landing platform *r*(*t*) = *V*(*t*)/*y*(*t*).

#### Estimation of set-points of relative rate of expansion

From the temporal dynamics of relative rate of expansion *r*(*t*) and using the algorithm from Goyal et al., (2021a), we identified the track segments in which bumblebees kept the relative rate of expansion close to constant (Figures 2C–2F). We refer to these segments as constant-*r* segments and characterize them with the average values of their state variables (*y*^∗^,*V*^∗^,*A*^∗^,*r*^∗^). We refer to *r*^∗^ as a set-point of relative rate of expansion that the bumblebees possibly aim to reach and fly at using their sensorimotor control system (Goyal et al., 2021a).

The output of the set-point detection algorithm used to identify constant-*r* segments depends on a threshold factor *f* that limits the variation around the mean of six linear regression parameters in terms of number of scale parameter of a *t*-distribution (Goyal et al., 2021a). This is similar to the number of standard deviations around the mean of a normally distributed variable. Higher *f* leads to more and wider constant-*r* segments, but also increases the possibility of detecting false-positives (Goyal et al., 2021a). We performed a sensitivity analysis by systematically varying the factor *f* in the range of 0.25 to 2.5, and assessed its effect on all our results (Figure S1). We found that the minimum threshold factor that gives robust results is *f* = 1.5, and therefore we here present the results for this threshold factor. For *f* = 1.5, the set-point detection algorithm identified 9,957 constant-*r* segments within 6,221 landing maneuvers out of a total of 10,005 maneuvers.

#### Extraction of entry segments (track segments leading up to constant-*r* segments)

During their landing maneuver, bumblebees hold the relative rate of expansion constant only for brief periods of time (Goyal et al., 2021a). To analyze their underlying closed-loop sensorimotor control system, we extracted the track segments leading up to these brief constant-*r* segments. Such segments are hereafter referred to as entry segments, as they potentially correspond to the moments when a bumblebee uses its sensorimotor control system to regulate relative rate of optical expansion and reach the desired set-point. See Methods S1 for the algorithm that we developed for identifying these entry segments.

For each constant-*r* segment, we identified the corresponding entry segment if there was a monotonic variation (either increase or decrease) of relative rate of expansion in the track segment before the constant-*r* segment (Figures 2C–2H). For the purpose of our analysis, the entry segment starts where the monotonic variation of relative rate of expansion starts or from *r* = 0.5 s^−1^ (a low value of relative rate of expansion), whichever occurs later in time. The entry segment ends where the constant-*r* segment begins.

Note that not all of the constant-*r* segments were linked to a respective entry segment. This is because either the complete width of a constant-*r* segment had not been captured with the choice of factor *f*, or due to oscillations in *r*, the monotonic variation was absent before constant-*r* segments. For factor *f* = 1.5, our entry-segment detection procedure was able to link 2,776 out of 9,957 constant-*r* segments with corresponding entry segments. Thus, because we use thousands of landing maneuvers, our relatively stringent detection algorithm for detecting entry segments does not restrict us in accurately describing the closed-loop dynamics of sensorimotor control system of the landing bumblebees.

#### Transfer-function based system identification

To find out if bumblebees regulate relative rate of optical expansion to reach the identified set-points, we analyzed the dynamics of all identified entry segments in conjunction with their corresponding constant-*r* segments. Specifically, we tested if the variation of relative rate of expansion (*r*) with time (*t*) during the entry segments can be captured as transient phases of a dynamic system aimed at reaching its steady-state – the corresponding set-point of relative rate of expansion (*r*^∗^).

We used a transfer-function based black-box system identification method (Ljung, 1999; Ogata, 2010) to find a dynamic system that can capture the variation of relative rate of expansion (*r*) with time (*t*) in each combined pair of entry segment and constant-*r* segment. Note that, we denote the *concatenated* temporal variation of *r* in both segments as *r*_c_(*t*). Using system identification, we aim to capture only the ‘mean variation’ in *r*_c_(*t*) and not the oscillations around it. This mean variation is characterized by the low-frequency content in the *r*_c_(*t*) signal (Figure 2C). Therefore, we filtered each *r*_c_(*t*) using a second-order two-directional Butterworth filter (*filtfilt* in Matlab 2020a) with a cut-off frequency of 5 Hz, resulting in the *filtered* temporal variation of optic expansion rate *r*_f_(*t*). Note that the high frequency content is likely a result of noise in the tracking measurements, or due to oscillations identified in a *r*-based control scheme (Croon and De Croon, 2016).

For the black-box system identification method, the set-point value *r*^∗^ acted as input to the dynamic system. It was constant over time and denoted as *r*^∗^(*t*) (Figure 1C). The desired variation of relative rate of expansion *r*_f_(*t*) that needed to be captured using the dynamic system was used as its output. A system identification process then involves parametrization of the dynamic system into a model structure of selected order, and estimation of these parameters using optimization over the defined input-output data (Ljung, 1999).

We used a transfer-function based black-box system identification method which characterizes a dynamic system as a system of linear time-invariant differential equations (Ljung, 1999). The coefficients of derivatives in these differential equations are parameters that are estimated using an iterative search algorithm that minimizes a quadratic prediction error criterion. This approach works in the frequency domain where such differential equations are represented as ratio of output to input polynomials. These polynomials are expressed as a function of a Laplacian variable *s*, and the ratio of these polynomials form a transfer function of the dynamic system.

We used a standard algorithm available in Matlab (v. 2020a, function *tfest*) to estimate a transfer function (Equation 1) from time-domain descriptions of input (*r*^∗^(*t*)) and output (*r*_f_(*t*)) signals. For each combination of *r*^∗^(*t*) and *r*_f_(*t*), we identified transfer functions of the first order to third order. In our results, we use second order transfer functions which are parameterized as

(Equation 1) $$\begin{array}{l} \frac{r_{\text{f}}\left(s\right)}{r^{*}\left(s\right)}=\frac{K\;w^{2}}{s^{2}+2Dws+w^{2}}\text{,} \end{array}$$

where *r*^∗^(*s*) and *r*_f_(*s*) are Laplace transforms of input *r*^∗^(*t*) and output *r*_f_(*t*), respectively; *K* (gain), *D* (damping ratio), and *w* (natural frequency) are parameters whose values are identified using system identification. The identified transfer functions were then simulated to produce the so-called *simulated* relative rate of expansion *r*_s_(*t*). Consequently, to select the model order, we computed fit-percentage *F* by comparing the model output (*r*_s_(*t*)) with the measured output *r*_f_(*t*) as

(Equation 2) $$F=100\%\;\left(1-\;\frac{\|r_{\text{f}}\left(t\right)-\;r_{\text{s}}\left(t\right)\|}{\|r_{\text{f}}\left(t\right)-\text{mean}\left(r_{\text{f}}\left(t\right)\right)\|}\right),$$

where $\left\|{\text{r}}_{\text{f}}\left(t\right)-r_{\text{s}}\left(t\right)\right\|$ and $\left\|{\text{r}}_{\text{f}}\left(t\right)-\text{mean}\left(r_{\text{f}}\left(t\right)\right)\right\|$ indicate the Euclidean norm of the time-series signals. The fit-percent *F* can vary between −∞ (bad fit) to 100 (perfect fit); a value of zero indicates that the model is no better than a straight line equal to the mean of *r*_f_(*t*).

#### Characterization of entry segments

For our modelling purpose, we assume that bumblebees keep the derivative of relative rate of expansion in each entry segment approximately constant. We refer to this constant as expansion-acceleration ($\dot{r_{\text{e}}}$) and estimate it from the linear regression *r*(*t*) = $\dot{r_{\text{e}}}$ *t* + *c* + ε (where *c* and ε denote intercept and residuals, respectively).

We tested this assumption by calculating the coefficient of determination (*R*^2^) for the aforementioned linear regression in each entry segment, which was very high (*R*^2^ = 0.980 [0.961 0.990], median [interquartile range]). This also holds for all tested treatments and both landing types (Figure S2). Moreover, the difference between the actual flight distance covered and the analytically computed flight distance if the bumblebees had performed the motion exactly at the estimated expansion-acceleration (Δ*d*) within the identified entry segments was also very low (Δ*d* = 0.7 mm [-1.0 mm, 2.6 mm], median [interquartile range]). Thus, the motion of landing bumblebees during the entry segments can be well approximated by a motion at a constant expansion-acceleration.

In addition to the slope of relative rate of expansion $\dot{r_{\text{e}}}$, we also identify the following three variables that are associated with an entry segment (Figure 2G): the set-point value that a bumblebee aspires to reach during the entry segment (*r*^∗^), the relative rate of expansion and distance from the platform at the start of the entry segment (*r*_0_ and *y*_0_, respectively), and the change in relative rate of expansion that is required during an entry segment (Δ*r*_e_ = *r*^∗^–*r*_0_).

We use the slope of relative rate of expansion (expansion-acceleration $\dot{r_{\text{e}}}$) as a performance measure for the closed-loop sensorimotor control system of bumblebees during landing. At the moment of switching the set-point, bumblebees can vary this slope depending upon the new set-point itself (*r*^∗^), the required step towards the new set-point (Δ*r*_e_) and the distance between the animal and the landing platform (*y*_0_). Bumblebees are expected to exhibit higher slopes for higher set-points, larger step-changes in *r* and when they are closer to the landing surface. This is because higher slopes will enable them to reach the desired set-point, and eventually the landing surface, more quickly. Additionally, this response rate $\dot{r_{\text{e}}}$ can also potentially vary with the environmental light intensity, optical expansion information available from the surface (landing patterns), or between the landings from free-flight and after take-off (the landing type).

In an entry segment, bumblebees either increase or decrease their relative rate of expansion to reach their set-point. As bumblebees advance towards the landing surface, the only way bumblebees can reduce their relative rate of expansion during an entry segment is by reducing their approach velocity, for example by decelerating. On the other hand, to increase their relative rate of expansion during an entry segment, bumblebees can potentially choose from several possibilities: fly at a constant approach velocity, weakly decelerate while approaching the platform, accelerate towards the landing surface or use a combination of these.

To find out how bumblebees use the transient response of their sensorimotor control system to advance towards the landing surface, we compute the mean acceleration ${\bar{A}}_{\text{e}}$ during each entry segment (Figure 2H). This mean acceleration equals the ratio of change in approach velocity Δ*V* that occurred in an entry segment and the corresponding time duration of the entry segment Δ*t* as ${\bar{A}}_{\text{e}}$ = Δ*V/*Δ*t*. Note that the positive value of mean acceleration in an entry segment occurs for segments in which bumblebees only accelerate towards the platform (Figure 2D) or accelerate more than they decelerate (Figure 2F).

In summary, we computed five parameters for each entry segment: ($\dot{r_{\text{e}}}$, *r*^∗^, Δ*r*_e_, *y*_0_, ${\bar{A}}_{\text{e}}$). Out of these, the four variables $\dot{r_{\text{e}}}$, *r*^∗^, Δ*r*_e_, *y*_0_ together completely characterize the motion at a constant expansion-acceleration, and ${\bar{A}}_{\text{e}}$ is the mean acceleration of such a motion.

#### Governing equations for motion at a constant expansion-acceleration

For an animal approaching a landing platform at time *t* (Figure 2B), we denote its distance to the platform as *y*(*t*), approach velocity as *V*(*t*), relative rate of optic expansion as *r*(*t*), approach acceleration as *A*(*t*), and following holds by definition:

(Equation 3A) $$V\left(t\right)=\frac{-dy\left(t\right)}{dt}\text{,}$$

(Equation 3B) $$A\left(t\right)=\frac{dV\left(t\right)}{dt}\text{,}$$

(Equation 3C) $$r\left(t\right)=\frac{V\left(t\right)}{y\left(t\right)}\text{.}$$

Differentiating Equation 3C with respect to time *t* results in the expansion acceleration

(Equation 4) $$\dot{r}=\frac{dr\left(t\right)}{dt}=\frac{y\left(t\right)A\left(t\right)+V^{2}\left(t\right)}{y^{2}\left(t\right)}\text{.}$$

Solving for approach acceleration *A*(*t*) in Equation 4 results in

(Equation 5) $$A\left(t\right)=\dot{r}y\left(t\right)-\frac{V^{2}\left(t\right)}{y\left(t\right)}\text{.}$$

During an entry segment, the motion of an animal towards the landing surface can be well approximated by a motion at a constant expansion-acceleration $\dot{r}$ (Figure S2A). Therefore, such a motion can be described by the following system of equations:

(Equation 6A) $$V\left(t\right)=\frac{-dy\left(t\right)}{dt}\text{,}$$

(Equation 6B) $$A\left(t\right)=\dot{r}y\left(t\right)-\frac{V^{2}\left(t\right)}{y\left(t\right)}\text{,}$$

(Equation 6C) $$y\left(t_{0}\right)=y_{0}\text{,}$$

(Equation 6D) $$V\left(t_{0}\right)=\left(r^{*}-\text{Δ}r_{\text{e}}\right)y_{0}\text{,}$$

where *r*^∗^ and Δ*r*_e_ are the new set-point and the required step-change in relative rate of expansion at the moment of switching the set-point *t* = *t*_0_ (Figure 2G). An example of a simulation of a motion at constant expansion acceleration along with the experimental data during an entry segment is shown in Figure S10.

### Quantification and statistical analysis

Using linear mixed-effects statistical models, we tested how expansion-acceleration $\dot{r_{\text{e}}}$ and the resulting mean acceleration ${\bar{A}}_{\text{e}}$ in an entry segment varies with the factors that can potentially influence them. For this, we developed two linear mixed-effects statistical models using the *lmer* package in R 4.0.2 (R Foundation). The dependent variables in the two models are the expansion-acceleration $\dot{r_{\text{e}}}$ and the resulting mean acceleration ${\bar{A}}_{\text{e}}$, respectively. In both models, the independent parameters are the starting distance from the landing surface (*y*_0_), the required step-change in relative rate of expansion (Δ*r*_e_), the final set-point to reach (*r*^∗^), landing patterns (checkerboard and spoke), environmental light intensities, and the starting condition of the landing maneuver (whether the landing is from a free-flight or after a take-off).

For each model, we first constructed a full model with aforementioned variables along with their interactions as fixed factors, and as random factors the experimental day, the landing approach number and the landing side (whether the landing disc is located on the hive side or on the food source side). We then used model dredging to find the minimal linear mixed-effects model. The model dredging revealed that the landing patterns did not affect either of the response variables ($\dot{r_{\text{e}}}$ or ${\bar{A}}_{\text{e}}$). Moreover, among all interaction terms, only the interaction log(*y*_0_) × log(Δ*r*_e_) was significant. As a result, both linear mixed-effects models reduced to the final version of

(Equation 7) $$\log\left(M_{i,d,a,s}\right)∼N\left(\text{α}+{\text{α}}_{d}+{\text{α}}_{a}+{\text{α}}_{s}+{\text{β}}_{1}\;\log\left(y_{0\;i,d,a,s}\right)+{\text{β}}_{2}\;{\text{MeduimLight}}_{i,d,a,s}+{\text{β}}_{3}\;{\text{SunriseLight}}_{i,d,a,s}+{\text{β}}_{4}\;{\text{fromTakeoff}}_{i,d,a,s}+{\text{β}}_{5}\;\log\left(\text{Δ}r_{\text{e}\;i,d,a,s}\right)+{\text{β}}_{6}\;\log\left(r_{i,d,a,s}^{*}\right)+{\text{β}}_{7}\;\log\left(y_{0\;i,d,a,s}\right)\times \log\left(\text{Δ}r_{\text{e}\;i,d,a,s}\right),{\text{σ}}^{2}\right)\text{,}$$

where *N* is the linear mixed-effects model function and *M* is either $\dot{r_{\text{e}}}$ or ${\bar{A}}_{\text{e}}$ for the two models, respectively. The subscripts define the entry segment number within the landing approach (*i*), the day (*d*), the landing approach number (*a*), and the landing side (*s*). The parameter α is the regression intercept for the twilight and free-flight starting condition (overall intercept), and α_*d*_, α_*a*_ and α_*s*_ are the day-specific, landing-approach-specific and landing-side-specific intercepts, respectively. MediumLight_*i,d,a,s*_, SunriseLight_*i,d,a,s*_ and fromTakeoff_*i,d,a,s*_ indicate if medium light condition, sunrise light condition and take-off starting condition are present (yes/no) for entry segment *i* from day *d*, landing approach number *a* and landing side *s*, respectively. Parameters ${\text{β}}_{k}\forall k\in \left\{2,3,4\right\}\;$ represent differences of fixed-effects from the overall intercept, and ${\text{β}}_{k}\forall k\in \left\{1,5,6,7\right\}$ represent the slopes of different covariates and the interaction (*k* = 7). Parameter σ is the standard deviation of the residual. We used Bonferroni correction to adjust significance values during post-hoc tests. The results for the $\dot{r_{\text{e}}}$ model and ${\bar{A}}_{\text{e}}$ model are provided in Tables 1 and 2, respectively.

## Data Availability

• This study uses the public database of landing maneuvers of bumblebees available as Mendeley Data: https://doi.org/10.17632/rrbjyhkm8z.1 (see key resources table). • The code used for the analysis is available as Methods S1 in a GitHub repository at https://github.com/kaku289/nimble-bbee-analysis/tree/entryTransients (see key resources table). • Any additional information required to reanalyze the data reported in this paper is available from the lead contact upon request.
