# Supplementary material for: Bumblebees land rapidly by intermittently accelerating and decelerating toward the surface during visually guided landings
Source: iScience. 2022 Apr 16;25(5):104265. doi: 10.1016/j.isci.2022.104265 (PMC9065724; doi:10.1016/j.isci.2022.104265)
Supplement: Document S1. Figures S1–S10 [file mmc1.pdf]

iScience, Volume *25*

Supplemental information

Bumblebees land rapidly by intermittently accelerating and decelerating toward the surface during visually guided landings

Pulkit Goyal, Johan L. van Leeuwen, and Florian T. Muijres

S1 Figures

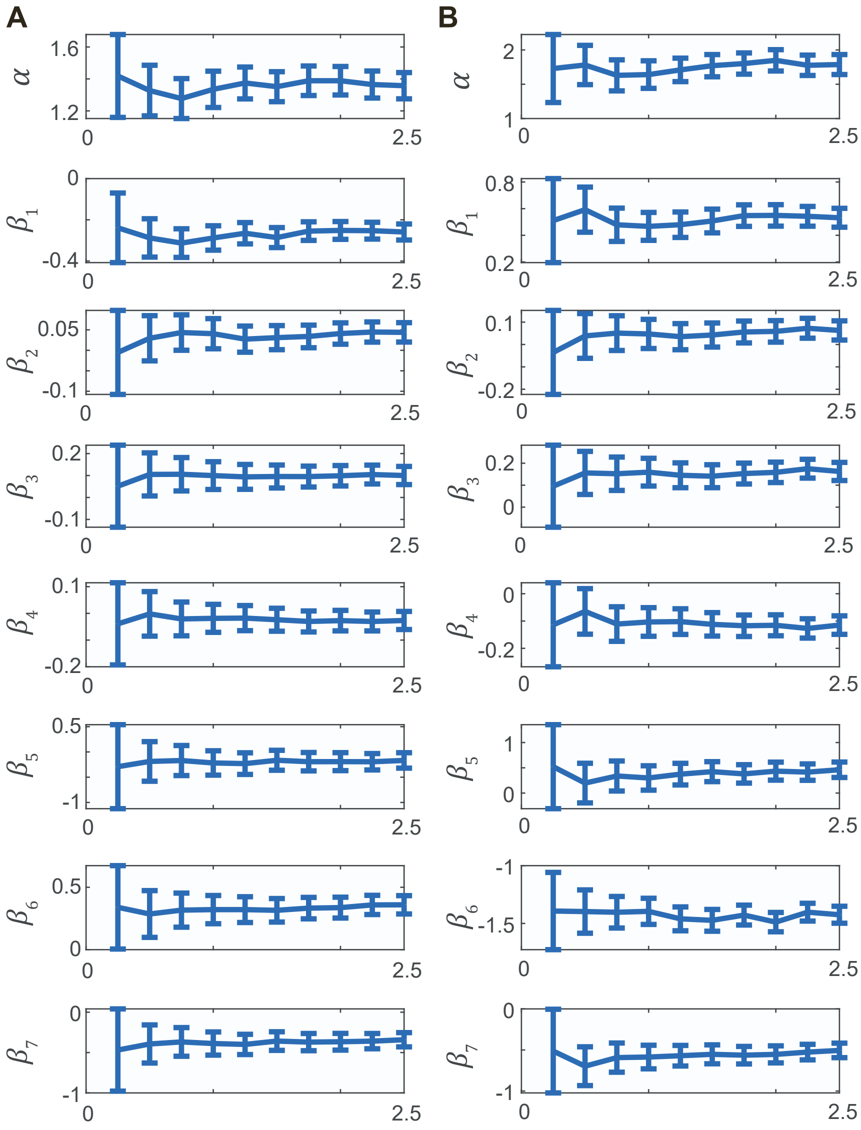

Figure S1: The effect of factor f on the results (related to Figures 4 and 5). (A,B) The dependence of expansion-acceleration $\dot{r}_e$ (A) and mean acceleration $\overline{A}_e$ (B) on distance from the landing surface (y_0), step-change of relative rate of expansion required in an entry segment Δr_e, the set-point r^*, environmental light intensity and landing type (landing after a take-off or from a free-flight) for different factors f $(\log(\dot{r}_e) \sim N(\alpha + \alpha_d + \alpha_a + \alpha_s + \beta_1\ \log(y_{0\ i,d,a,s}) + \beta_2\ \mathrm{MEDIUMlight}_{i,d,a,s} + \beta_3\ \mathrm{SUNRISElight}_{i,d,a,s} + \beta_4\ \mathrm{fromTakeoff}_{i,d,a,s} + \beta_5\ \log(\Delta r_{e\ i,d,a,s}) + \beta_6\ \log(r^*_{i,d,a,s}) + \beta_7\ \log(y_{0\ i,d,a,s}) \times \log(\Delta r_{e\ i,d,a,s}),\ \sigma^2)$, similar equation holds for $\overline{A}_e$). The vertical bars for each coefficient indicate 95% confidence intervals.

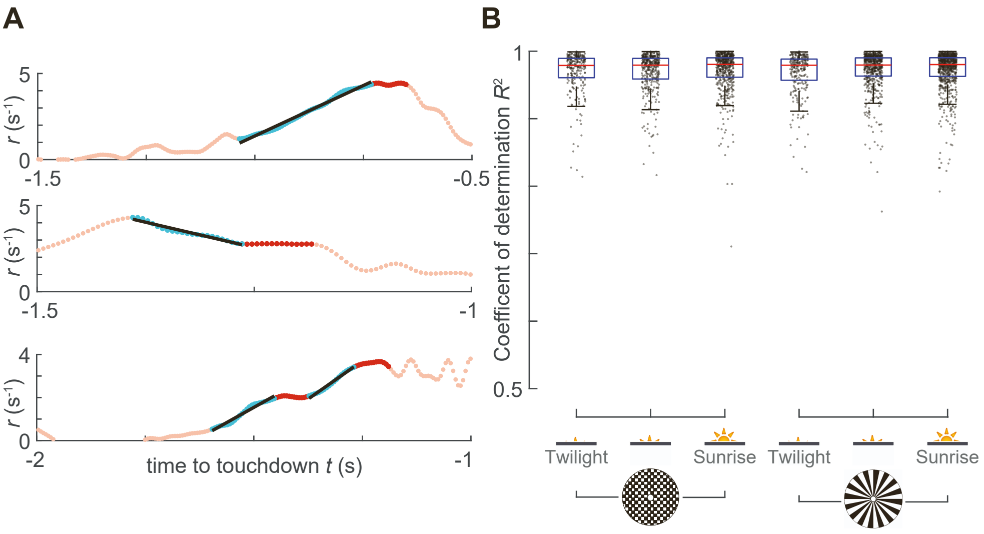

Figure S2: During the transient phases of a landing approach, bumblebees keep the expansion acceleration $\dot{r}_e$ approximately constant (related to Figures 2 and 4). (A) Three examples of landing approaches in which the variation of relative rate of expansion r during entry segments (blue) is fitted with time-to-touchdown t using a linear regression (black). This linear regression approximates the optic expansion acceleration with a constant value. The constant-r segment is shown in red and the black arrow indicates the variation of abscissa data as a bumblebee approaches the landing disc. (d) The goodness of fit of the linear regression model for all identified transient phases, in the six experimental conditions, as defined by the coefficient of determination (R^2). At each condition, we show a box plot and the coefficient of determination (R^2) at all transient phases (dots). The median coefficient of determination is more than 0.98 in all tested treatments.

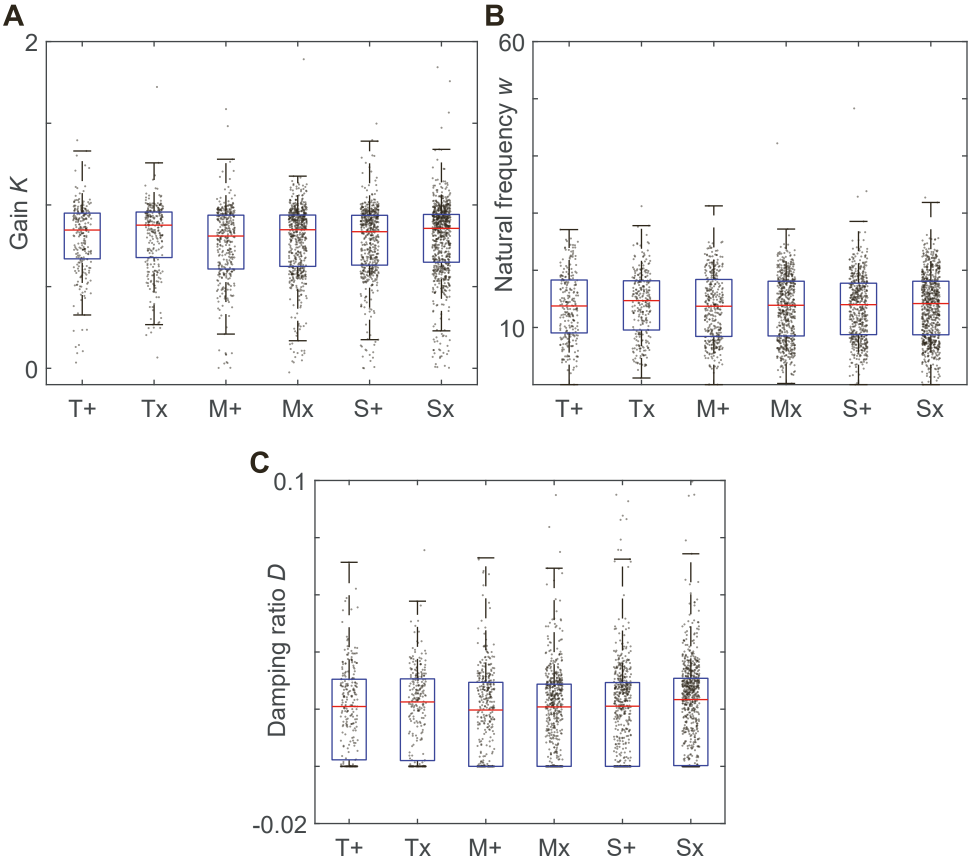

Figure S3: The parameters identified using system identification (related to Figure 3). (a) Gain K, (b) natural frequency w and (c) damping ratio D. For each treatment, the data from different landing types (landings initiated from free-flight or take-off) are shown together. The blue box indicates interquartile range, and black lines indicate the maximum and minimum, respectively. The values that lie 1.5 times the interquartile range away from the top or the bottom of the blue box are labelled as outliers (twilight (T), medium (M) and sunrise (S) light conditions, checkerboard (+) and spoke (x) landing patterns).

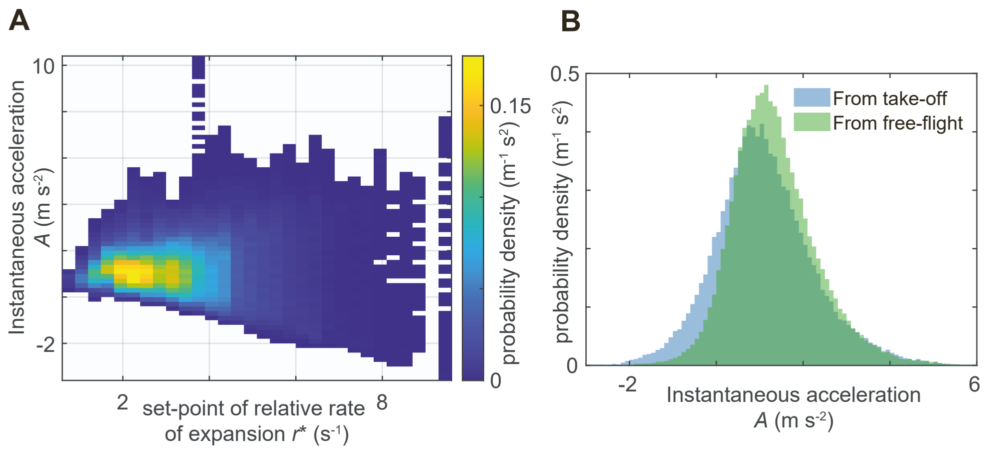

Figure S4: The variation of instantaneous acceleration $A(t)$ during entry segments with the set-point of relative rate of expansion r^* and landing type (landing from a free-flight or after take-off) (related to Figure 5). The data plotted here corresponds to 2,620 entry segments in which bumblebees exhibited positive mean acceleration ($\bar{A}_e > 0$).

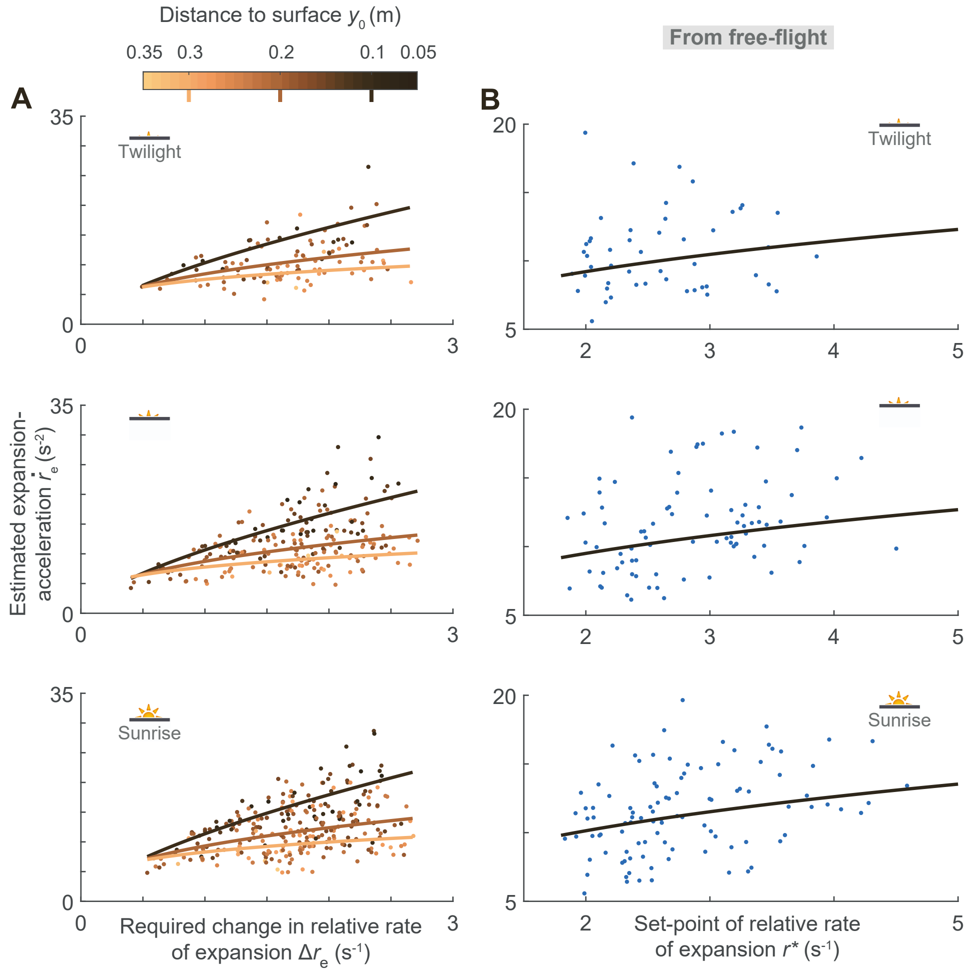

Figure S5: The depiction of how bumblebees modulate their sensorimotor control response $\dot{r}_e$ in different light conditions when they landed from free-flight (related to Figure 4). (A) Effect of required step-change in relative rate of expansion in an entry segment Δr_e and distance from the landing platform y_0 on $\dot{r}_e$, data points are shown for $r^* \in [2.28, 3.28]\ s^{-1}$, solid curves depict statistical model output, and are plotted for $r^* = 2.78\ s^{-1}$ (the median value). (B) Effect of set-point of relative rate of expansion r^* on $\dot{r}_e$, data points are plotted for $\Delta r_e \in [1.28, 2.08]\ s^{-1}$ and $y_0 \in [0.18, 0.24]\ m$ (these intervals are centered around their median values). See Table ?? for statistical model output. (A,B) Top, middle and bottom panels correspond to low, medium and high light conditions, respectively.

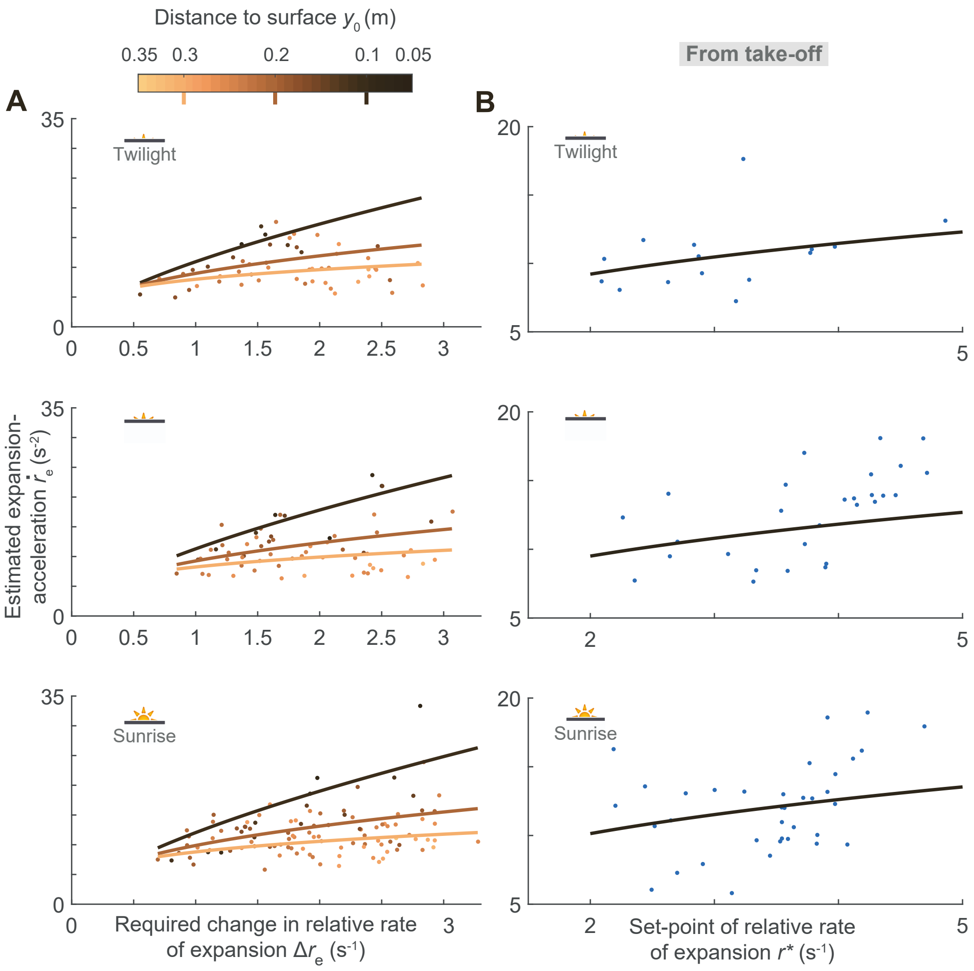

Figure S6: The depiction of how bumblebees modulate their sensorimotor control response $\dot{r}_e$ in different light conditions when they landed after take-off (related to Figure 4). (A) Effect of required step-change in relative rate of expansion in an entry segment Δr_e and distance from the landing platform y_0 on $\dot{r}_e$, data points are shown for $r^* \in [2.28, 3.28]\ s^{-1}$, solid curves depict statistical model output, and are plotted for $r^* = 2.78\ s^{-1}$ (the median value). (B) Effect of set-point of relative rate of expansion r^* on $\dot{r}_e$, data points are plotted for $\Delta r_e \in [1.28, 2.08]\ s^{-1}$ and $y_0 \in [0.18, 0.24]\ m$ (these intervals are centered around their median values). See Table ?? for statistical model output. (A,B) Top, middle and bottom panels correspond to low, medium and high light conditions, respectively.

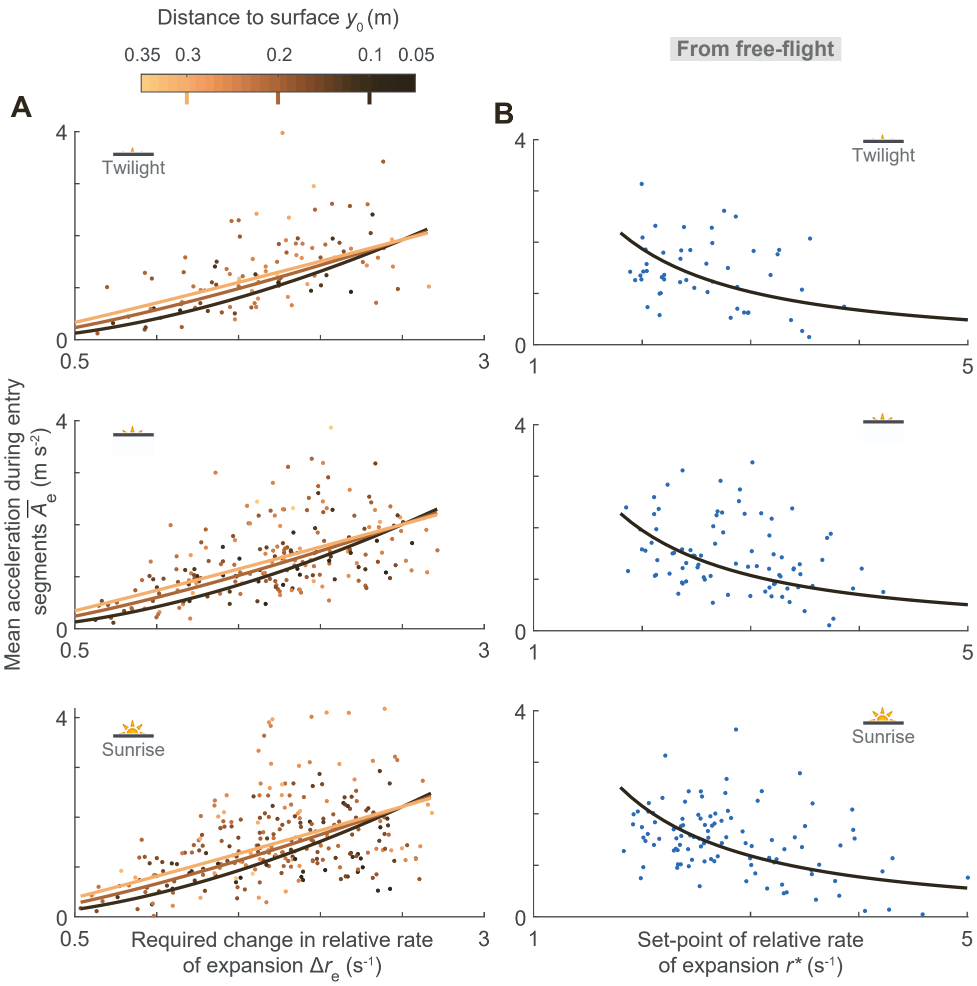

Figure S7: The depiction of how bumblebees modulate their mean acceleration $\overline{A}_e$ during an entry segment in different light conditions when they landed from free-flight (related to Figure 5). (A) Effect of required step-change in relative rate of expansion in an entry segment Δr_e and distance from the landing platform y_0 on $\overline{A}_e$, data points are shown for $r^* \in [2.28, 3.28]\ s^{-1}$, solid curves depict statistical model output, and are plotted for $r^* = 2.78\ s^{-1}$ (the median value). (B) Effect of set-point of relative rate of expansion r^* on $\dot{r}_e$, data points are plotted for $\Delta r_e \in [1.28, 2.08]\ s^{-1}$ and $y_0 \in [0.18, 0.24]\ m$ (these intervals are centered around their median values). See Table **??** for statistical model output. (A,B) Top, middle and bottom panels correspond to low, medium and high light conditions, respectively.

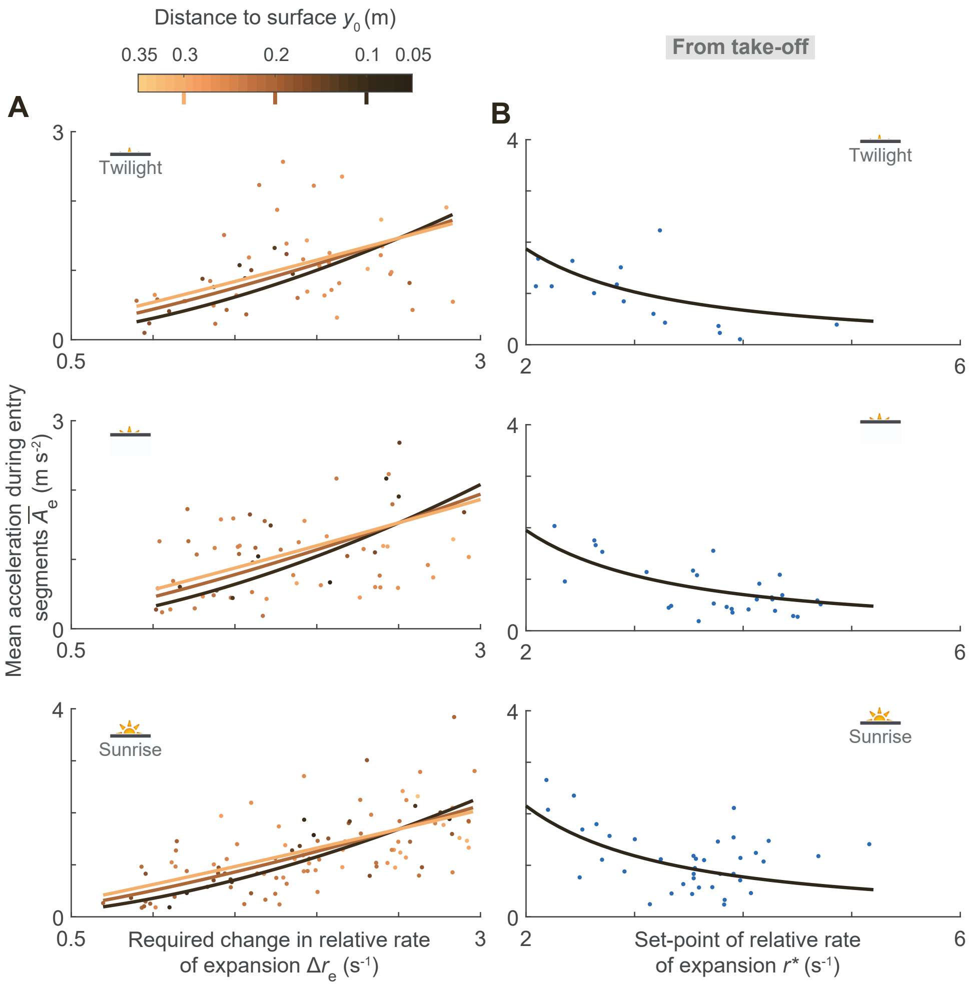

Figure S8: The depiction of how bumblebees modulate their mean acceleration $\overline{A}_e$ during an entry segment in different light conditions when they landed after take-off (related to Figure 5). (A) Effect of required step-change in relative rate of expansion in an entry segment Δr_e and distance from the landing platform y_0 on $\overline{A}_e$, data points are shown for $r^* \in [2.28, 3.28]\ s^{-1}$, solid curves depict statistical model output, and are plotted for $r^* = 2.78\ s^{-1}$ (the median value). (B) Effect of set-point of relative rate of expansion r^* on $\dot{r}_e$, data points are plotted for $\Delta r_e \in [1.28, 2.08]\ s^{-1}$ and $y_0 \in [0.18, 0.24]\ m$ (these intervals are centered around their median values). See Table **??** for statistical model output. (A,B) Top, middle and bottom panels correspond to low, medium and high light conditions, respectively.

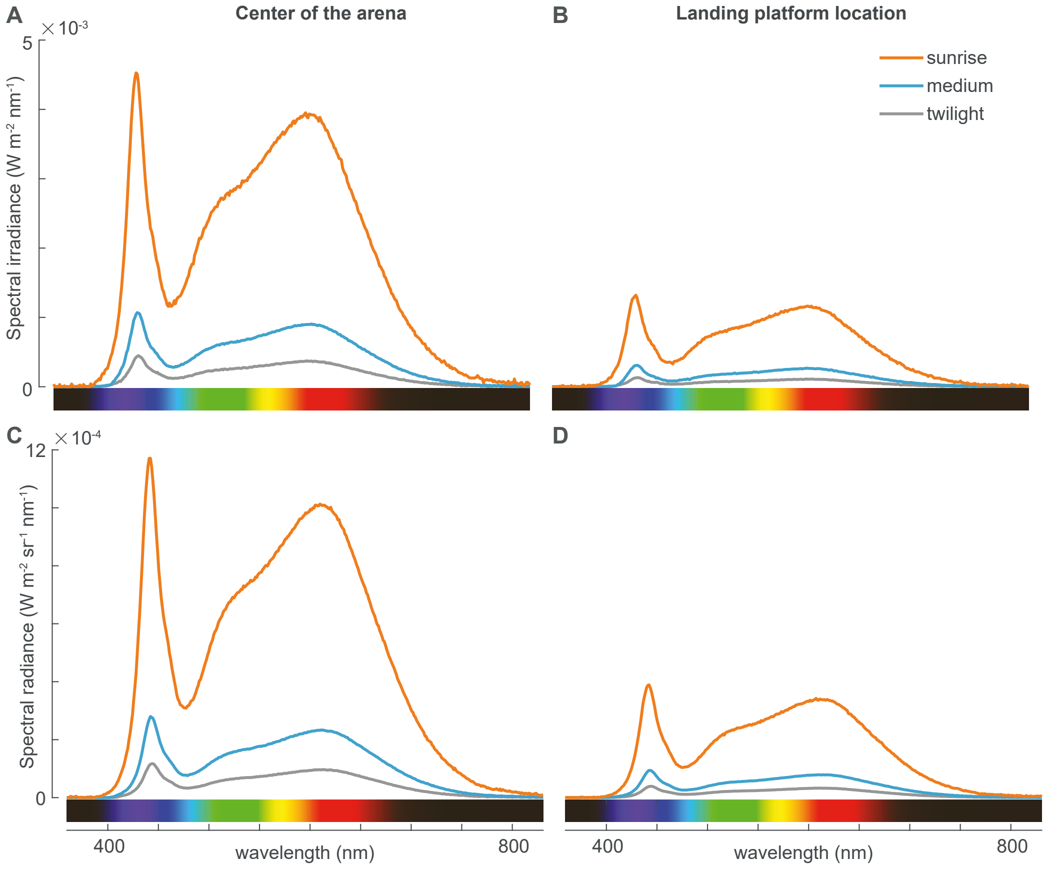

Figure S9: Light intensities for the twilight (gray), medium (blue) and sunrise (orange) light conditions (related to Figure 2). Spectral irradiance (A) and spectral radiance (C) at the center of the flight arena. Spectral irradiance (B) and spectral radiance (D) at the centre of the landing platforms.

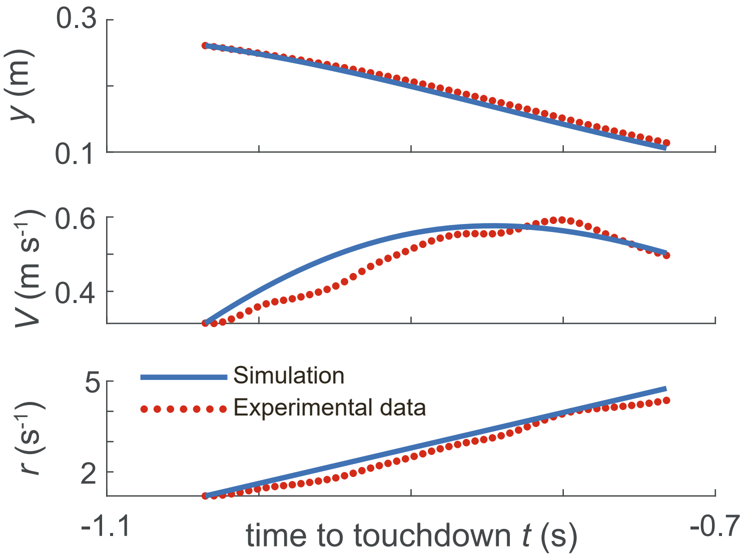

Figure S10: A simulation of the motion at constant expansion acceleration along with the experimental data during an entry segment (related to Figures 2 and 4). The variation of approach distance y (top), approach velocity V (middle) and relative rate of expansion r (bottom) with time-to-touchdown t for an entry segment identified during a landing approach (shown in Figure S2A). The simulation is carried out for $y_0 = 0.261\ m,\ t_0 = -1.035\ s,\ r^* = 4.39\ s^{-1},\ \Delta r_e = 3.18\ s^{-1}$ and $\dot{r} = 11.72\ s^{-2}$.
